# What is the optimum time to start antiretroviral therapy in people with HIV and tuberculosis coinfection? A systematic review and meta‐analysis

**DOI:** 10.1002/jia2.25772

**Published:** 2021-07-21

**Authors:** Rachael M Burke, Hannah M Rickman, Vindi Singh, Elizabeth L Corbett, Helen Ayles, Andreas Jahn, Mina C Hosseinipour, Robert J Wilkinson, Peter MacPherson

**Affiliations:** ^1^ Malawi‐Liverpool‐Wellcome Clinical Research Programme Blantyre Malawi; ^2^ Clinical Research Department Faculty of Infectious and Tropical Disease London School of Hygiene and Tropical Medicine London UK; ^3^ Department HIV, Hepatitis and STIs World Health Organisation Geneva Switzerland; ^4^ Zambart Lusaka Zambia; ^5^ Department of HIV and AIDS Ministry of Health Malawi Lilongwe Malawi; ^6^ International Training and Education Center for Health Department of Global Health University of Washington Seattle WA USA; ^7^ University North Carolina Chapel Hill NC USA; ^8^ UNC Project Lilongwe Malawi; ^9^ Dept Infectious Disease Imperial College London London UK; ^10^ Wellcome Centre for Infectious Diseases Research in Africa and Institute of Infectious Disease and Molecular Medicine University of Cape Town Observatory Republic of South Africa; ^11^ Francis Crick Institute London UK; ^12^ Department of Clinical Sciences Liverpool School of Tropical Medicine Liverpool UK

**Keywords:** tuberculosis, HIV, antiretroviral therapy, systematic review, public health, rapid ART

## Abstract

**Background:**

HIV and tuberculosis are frequently diagnosed concurrently. In March 2021, World Health Organization recommended that antiretroviral therapy (ART) should be started within two weeks of tuberculosis treatment start, at any CD4 count. We assessed whether earlier ART improved outcomes in people with newly diagnosed HIV and tuberculosis.

**Methods:**

We did a systematic review by searching nine databases for trials that compared earlier ART to later ART initiation in people with HIV and tuberculosis. We included studies published from database inception to 12 March 2021. We compared ART within four weeks versus ART more than four weeks after TB treatment, and ART within two weeks versus ART between two and eight weeks, and stratified analysis by CD4 count. The main outcome was death; secondary outcomes included IRIS and AIDS‐defining events. We pooled effect estimates using random effects meta‐analysis.

**Results and discussion:**

We screened 2468 abstracts, and identified nine trials. Among people with all CD4 counts, there was no difference in mortality by earlier ART (≤4 week) versus later ART (>4 week) (risk difference [RD] 0%, 95% confidence interval [CI] −2% to +1%). Among people with CD4 count ≤50 cells/mm^3^, earlier ART (≤4 weeks) reduced risk of death (RD −6%, −10% to −1%). Among people with all CD4 counts earlier ART (≤4 weeks) increased the risk of IRIS (RD +6%, 95% CI +2% to +10%) and reduced the incidence of AIDS‐defining events (RD −2%, 95% CI −4% to 0%). Results were similar when trials were restricted to the four trials which permitted comparison of ART within two weeks to ART between two and eight weeks. Trials were conducted between 2004 and 2014, before recommendations to treat HIV at any CD4 count or to rapidly start ART in people without TB. No trials included children or pregnant women. No trials included integrase inhibitors in ART regimens.

**Discussion:**

Earlier ART did not alter risk of death overall among people living with HIV who had TB disease. For logistical and patient preference reasons, earlier ART initiation for everyone with TB and HIV may be preferred to later ART.

## 1 Introduction

Tuberculosis (TB) is the most important cause of morbidity and mortality among people living with HIV (PLHIV) globally [1, 2]. All people with TB should be offered testing for HIV and everyone newly diagnosed with HIV should have TB screening, meaning that TB and HIV are often diagnosed simultaneously.

The potential risks of starting antiretrovial therapy (ART) early in TB treatment may include drug toxicity, drug–drug interactions [3], a perceived high pill burden for patients and immune reconstitution inflammatory syndrome (IRIS) [4, 5], all of which may affect adherence and retention in care [6]. The benefits of starting ART early in TB treatment may include reduced HIV complications (including progression to AIDS‐defining illness and death) [7, 8] and shorter time to HIV viral load suppression, which has benefits for patients and their partners through reduced HIV transmission [9]. Early ART initiation may also simplify programme implementation and avoid unintended delays due to pending CD4 count results and more complex differentiated pathways [10].

In March 2021, World Health Organization recommended that antiretroviral therapy (ART) should be started within two weeks of tuberculosis treatment start, at any CD4 count [11]. The previous (2016) WHO recommendations [12] were that people with CD4 < 50 cells/mm^3^ should start ART within two weeks and others to start ART within eight weeks. The old 2016 recommendation may have caused logistical complexity, particularly in situations where CD4 cell counts are not readily available. Furthermore, since 2017 rapid ART (within seven days of HIV diagnosis) has been recommended for most PLHIV without TB, as evidence suggests that reducing delay between diagnosis of HIV and starting ART improves outcomes [13, 14]. The old 2016 recommendation was produced before much of the evidence for rapid ART in people with HIV without TB. Prior to the March 2021 WHO guidelines, at least three high HIV burden countries (Malawi, Zambia and Uganda) had already issued national guidelines recommending ART within or at two weeks after starting TB treatment for people with TB and HIV at all CD4 cell counts [15, 16, 17].

In light of high levels of mortality associated with HIV and TB coinfection, and the trend towards earlier initiation of ART in PLHIV without TB, we sought to review the evidence around the timing of ART initiation PLHIV who have TB disease. This systematic review was conducted before the March 2021 updated guidelines were issued; this work was part of the evidence considered by the guideline development group.

## 2 Methods

### 2.1 Inclusion and exclusion criteria

We systematically reviewed studies that compared different time‐points of ART initiation in PLHIV who were not already on ART and who were initiating TB treatment. We included studies of PLHIV of any age, in any country setting. We included two sets of interventions and comparators. First, we compared starting ART within two weeks to starting ART between two and eight weeks after TB treatment as these are the two strategies recommended in 2016 WHO guidance (Comparison A). However, we included a comparison with a more general definition (Comparison B) because some trials used different time intervals to define early and delayed ART initiation (e.g. compared four weeks to twelve weeks). We analysed data across all CD4 cell counts, and by CD4 cell count strata using a cut‐point of CD4 count 50 cells/mm^3^ to reflect 2016 WHO guidelines. These categorisations were pre‐specified. The main outcome of interest was mortality; secondary outcomes included incidence of IRIS, AIDS‐defining events, serious adverse events (SAEs), viral load suppression and loss to follow‐up.

We only included randomized trials and we excluded trials that were solely conducted among people with TB meningitis, as TB meningitis is managed substantially differently from pulmonary TB (e.g. steroids are routinely used, patients would usually be hospital inpatients), and the risks associated with IRIS may be higher [18].

### 2.2 Search strategy and data extraction

The protocol and search strategy are available online at PROSPERO (CRD42020190396). We used a broad search strategy to identify randomized controlled trials that compared the timing of ART in people with HIV and TB. The search was designed with the assistance of a specialist librarian. We searched eight databases including Medline and Embase and two clinical trial registries for articles published between database inception and 12 March 2020 (Appendix S1).

All titles and abstracts were reviewed by RMB and HMR using the Rayaan software program [19]. After reviewing an initial 10% sample of titles/abstracts in duplicate, and determining that there was 100% agreement between reviewers, the remainder of title abstracts were reviewed by one reviewer only. All papers at full‐text review stage were reviewed by both RMB and HMR for decision on inclusion. Both RMB and HMR extracted data independently in duplicate from studies selected for inclusion. Data were extracted from published manuscripts, supplementary data files and study protocols and entered into a spreadsheet. Where CD4 cell count‐disaggregated data were not available, we contacted authors.

### 2.3 Statistical methods and risk of bias assessment

Data were pooled in meta‐analysis using package “meta” [20] in R [21]. We expressed effects as absolute risk differences. Random effects meta‐analysis was used because we anticipated heterogeneity in results between studies. We used the Mantel–Haenszel method to estimate confidence intervals (CI) of risk differences, and the DerSimonian Laird method to estimate variance of pooled effect [22]. We used an intention‐to‐treat approach and included all randomized participants in the denominator with the exception of one study, which reported outcomes for a pre‐specified modified intention‐to‐treat population where participants whose sputum samples were culture negative for *Mycobacterium tuberculosis* were excluded after randomization [23]. For the HIV viral suppression outcome, the denominator was the number of people with HIV viral load measured.

We used the Cochrane Risk of Bias 2 tool [24] to assess study quality. Risk of bias assessments were performed in duplicate by HMR and RMB and differences were resolved by consensus and discussion with PM. PRISMA guidelines were used for reporting (checklist in Appendix S2).

## 3 Results and discussion

**Figure 1:**
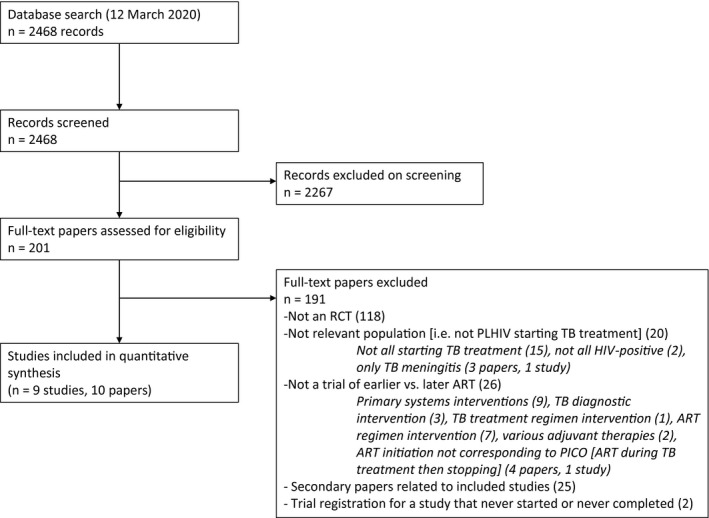
PRISMA diagram. PLHIV, people living with HIV; RCT, randomized controlled trial; TB, tuberculosis.

After removing duplicates, we identified 2468 articles, of which 10 were included in our quantitative synthesis and meta‐analysis (Figure 1). We identified nine studies that compared the effects of earlier to later ART initiation among PLHIV and TB disease, comprised of 10 manuscripts and one unpublished paper (identified in the search through an abstract presented at the IAS Conference 2016). We contacted the authors of the unpublished paper and received data to permit inclusion. These trials were conducted between June 2004 and December 2014 and included a total of 5030 randomized participants. Five studies were conducted in sub‐Saharan Africa, including two multi‐country trials; three were in Asia, and one study included participants from Africa, Asia, North America and South America.

The nine trials had important differences related to patient population (particularly CD4 count inclusion criteria), definitions of TB (whether people with probable TB, or with extrapulmonary TB were included) and timing of ART (Table 1). Four of the nine trials included participants with clinically diagnosed TB (i.e. TB diagnosed without microbiological confirmation) [25, 26, 27, 28]. Three studies restricted participation to those with pulmonary TB only [23, 29], whereas six trials included participants with pulmonary and extrapulmonary TB [25, 26, 27, 28, 30, 31]. No trials included children under 13 years of age: two trials included both adults and adolescents (age ≥13 years) [26, 30] and the remaining seven included adults (≥18 years) only.

**Table 1:** Characteristics of included studies

Trial	Setting and blinding (Country/countries, study years, blinding)	ART initiation interventions	Eligibility criteria and population			Number of participants	Outcomes	
			Participant criteria	CD4 cell count	TB disease definition		Study primary outcome	Secondary outcomes relevant to review
Shao et al, 2009 (THIRST)	Tanzania June 2004 to September 2007 Unblinded	Earlier ART: two weeks Later ART: eight weeks ART regimen: ABC/3TC/AZT	Age ≥13, ART‐naive, non‐pregnant, admitted to hospital, no very abnormal laboratory tests	Eligibility: Total lymphocyte count <1200 cells/mm^3^ (no CD4 criteria) Median (IQR) CD4 cell count (cells/mm^3^): 103 (55 to 155) CD4 < 50 cells/mm^3^: not stated	Eligibility: Smear‐positive pulmonary or extra‐pulmonary TB. CNS TB excluded. No sputum culture available	70 (all with death outcome at 104 weeks)	Rate of TB‐IRIS, measured up to 104 weeks	Mortality, IRIS, ADEs, LFTU, VL suppression
Abdool Karim et al, 2010; 2011 (SAPiT)^a^	South Africa June 2005 to July 2008 Unblinded	2011 report Earlier ART: Within four weeks (median time to ART 21 days, IQR 15 to 29 days) Later ART: Between eight and twelve weeks [median time to ART 97 days, IQR 77 to 126 days) 2010 report Earlier ART: During TB treatment (within four weeks and eight to twelve weeks) Later ART: 26 weeks (i.e. at end of TB treatment) ART regimen: ddI/3TC/EFV	Age >18, non‐pregnant (women on contraception), “ambulatory”, “absence of clinical contraindications to ART”	Eligibility: CD4 ≤500 cells/mm^3^ Median (IQR) CD4 cell count (cells/mm^3^): four‐week group, 154 (72‐261); eight‐ to twelve‐week group, 149 (77 to 244), 26‐week group 140 (69 to 247) CD4 < 50 cells/mm^3^: 17%	Eligibility: Positive sputum smear for acid‐fast bacilli Participants: 148/429 (35%) had previous TB, and were treated with “a 60‐day intensive regimen that included streptomycin”. 9/203 participants with sputum culture positive had MDR‐TB	2011 report 429 (321 have death outcome at 18 months) 2010 report 642 (includes the 429 in 2011 report) (523 had death outcome at 24 months)	2011 report Incidence of AIDS or death measured up to 18 months from enrolment (composite outcome) (hazards) 2010 report Death from any cause between enrolment and 1 September 2008 (median 12.1 months in trial)	Mortality, IRIS, ADEs, LFTU, VL suppression
Havlir et al, 2011 (STRIDE)	13 countries in four continents Sep 2006 to Aug 2009 Participants and people providing clinical care not blinded. Endpoint adjudicators blinded	Earlier ART: Within two weeks (median time to ART 10 days) Later ART: Between eight and twelve weeks (median time to ART 70 days) ART regimen: EFV/FTC/TDF (or appropriate substitution became pregnant during trial)	Age ≥13 years, non‐pregnant at recruitment, Karnofsky score ≥20, no known or suspected MDR TB,^b^ No very abnormal laboratory tests	Eligibility: CD4 ≤ 250 cells/mm^3^ Median (IQR) CD4 cell count (cells/mm^3^): 77 (36 to 145) CD4 < 50 cells/mm^3^:35%	Eligibility: Confirmed or probable TB at any site Participants: 46% confirmed TB, 53% probable TB 48/162 positive TB cultures were from non‐sputum sites 5/120 cultures with drug susceptibility testing were MDR‐TB, but no participants were initiated on MDR‐TB treatment initially (as known or suspected MDR‐TB was an exclusion criterion)	806 randomized (744 have death outcome recorded)	Proportion of patients who survived and did not have a new AIDS‐defining illness at 48 weeks	Mortality, IRIS, ADEs, LFTU, VL suppression
Blanc *et al*, 2011 (CAMELIA)	Cambodia Jan 2006 to May 2009 Unblinded	Earlier ART: two weeks Later ART: eight weeks ART regimen: d4t/3TC/EFV	Age ≥18, ART naive, non‐pregnant. Included both hospital inpatients and outpatients	Eligibility: CD4 ≤ 200 cells/mm^3^ Median (IQR) CD4 cell count (cells/mm^3^): 25 (10 to 56) CD4 < 50 cells/mm^3^: 71%	Eligibility: Smear‐positive for acid‐fast bacilli on sample from any site Participants: 33% extra‐pulmonary TB. Six and seven people in earlier and later ART arm, respsectively, had MDR‐TB on culture specimen. Not reported what TB treatment regimen was commenced for participants	661 randomized, (633 with death outcome recorded)	Survival (as a hazard), measured up to 50 weeks from enrolment of last participant (median 25 months)	Mortality, IRIS, SAEs, LFTU, VL suppression
Manosuthi *et al*, 2012 (TIME)	Thailand Oct 2009 to May 2011 Unblinded	Earlier ART: four weeks Later ART: 12 weeks ART regimen: TDF/3TC/EFV	Age ≥18, non‐pregnant, ART‐naive, not “moribund”, no very abnormal laboratory tests	Eligibility: CD4 <350 cells/mm^3^ Median (IQR) CD4 cell count (cells/mm^3^): 43 (47 to 106) CD4 < 50 cells/mm^3^: 54%	Eligibility: Confirmed or probable TB at any site Participants: 65% microbiologically confirmed TB, 53% extrapulmonary or disseminated TB. All participants started on RHZE regimen. Not reported whether any MDR‐TB	156 (all have death outcome)	All‐cause mortality at one year^c^	Mortality, IRIS, ADEs, LFTU
Sinha et al 2012	India Start not stated. Follow‐up ended March 2011. Unblinded	Earlier ART: Between two and four weeks Later ART: Between eight and twelve weeks ART regimen: d4T or AZT/3TC/EFV	Age ≥18, non‐pregnant, ART‐naïve, no “severe illness”, no very abnormal liver function	Eligibility: Any Median CD4 cell count (cells/mm^3^): Early group, 133 (range 7 to 588); later group 152 (range 14‐648) CD4 < 50 cells/mm^3^: not stated	Eligibility: Confirmed or probable TB at any site Participants: 77% extrapulmonary TB, not stated whether confirmed versus probable. Presence/absence MDR‐TB not reported NB. TB treatment is 3× week directly observed therapy, not daily	181 randomized (138 with mortality outcome)^d^	All‐cause mortality and ART failure (co‐primary outcomes) at 12 months. Reported as a hazard	Mortality, IRIS, ADEs, LFTU
Mfinanga et al 2014 (TB‐HAART)	South Africa, Tanzania, Uganda, Zambia Jan 2008 to April 2013 Double‐blinded placebo‐controlled until six months (end of TB treatment)	Earlier ART: two weeks Later ART: six months ART regimen: AZT/3TC/EFV (EFV switched to NVP if became pregnant during trial)	Age >−18, non‐pregnant, no WHO stage 4 condition, Weight >30 kg, No very abnormal laboratory tests	Eligibility: CD4 ≥ 220 cells/mm^3^ Median (IQR) CD4 cell count (cells/mm^3^): 367 (289 to 456) CD4 < 50 cells/mm^3^: None	Eligibility: Smear and culture‐positive drug‐sensitive pulmonary TB^(5)^	1675 randomized (1538 with death outcome)^e^	Composite: failure of TB treatment, TB recurrence, death within 12 months of starting TB treatment (proportion)	Mortality, IRIS, LFTU
Amogne et al 2015	Ethiopia June 2008 to April 2011 Unblinded	Earlier ART: one week (median time to ART seven days) Middle time point: four weeks (median time to ART 28 days) Later ART: eight weeks (median time to ART 56 days) ART regimen: EFV + 3TC + study site choice of AZT/TDF/d4T	Age ≥18, non‐pregnant, Karnofsky score ≥40, No very abnormal laboratory tests	Eligibility: CD4 < 200 cells/mm^3^ Median (IQR) CD4 cell count (cells/mm^3^): 67 (39 to 106) early group, 71 (45 to 121) middle group, 76 (47 to 110) later group CD4 < 50 cells/mm^3^: 31%	Eligibility: Confirmed or probable TB at any site, but excluding CNS TB (22) Participants: 26% had EPTB, 53% had smear‐negative TB. Presence or absence MDR‐TB not reported	478 randomized (432 with known mortality outcome)	All‐cause mortality at 48 weeks. Reported as a hazard	Mortality, ADEs, SAEs, IRIS, VL suppression
Merle et al 2020 (RAFA)	Guinea, Benin, Senegal May 2011 to Dec 2014 Unblinded	Earlier ART: two weeks Later ART: eight weeks ART regimen: EFV + 2 NRTIs according to national programmes	Age ≥18 and ≤65, ART naïve	Eligibility: CD4 ≥ 50 cells/mm^3^ Median (IQR) CD4 cell count (cells/mm^3^): 179 (IQR 101 to 288) early ART; 184 (108 to 272) late. CD4 < 50 cells/mm^3^: None	Eligibility: Microbiologically confirmed drug sensitive pulmonary TB	489 randomized into earlier versus later ART arms^f^ (474 with death recorded)	All‐cause mortality at 12 months. Reported as a hazard	Mortality, IRIS, LFTU, VL suppression

3TC, lamivudine; ABC, abacavir; ADEs, AIDS‐defining events; ART, anti‐retroviral therapy; AZT, zidovudine; d4T, stavudine; ddI, didanosine; DSMB, data safety and monitoring board; EFV, efavirenz; FTC, emtricitabine; IRIS, immune reconstitution inflammatory syndrome; LTFU, loss to follow‐up; MDR, multi‐drug resistant; NRTI, nucleoside reverse transcriptase inhibitor; NVP, nevirapine; SAEs, severe adverse events; TDF, tenofovir disoproxil fumarate; ULN, upper limit of normal; VL HIV‐1, viral load suppression.

^a^ SAPiT was designed as a three arm trial, starting ART at 4, 12 and 26 weeks. Part way through the trial, the DSMB recommended terminating the 26‐week arm and commencing everyone in the 26‐week arm on ART. The results are reported in two papers: 2010 paper compared the two earlier groups (four and twelve weeks) to the later 26‐week group. The 2011 SAPiT paper presented the results of the four‐week group compared to the twelve‐week group

^b^ although entry criteria were no known or suspected MDR TB (i.e. all participants were initially started on first line TB treatment), of 120 people with drug susceptibility testing available there were 5 people with MDR TB, 8 people with INH resistance and 3 people with single drug resistance of rifampicin, pyrazinamide or ethambutol

^c^ it is unclear whether the primary outcome is related to proportion of deaths, or hazard of death. Both are reported. Death up until two years is reported as a secondary outcome

^d^ participants excluded after randomization if they did not initiate ART at the protocol‐designated timepoints, due to being unable to initiate on time, not returning for initiation, or starting ART elsewhere. We have included all these participants in the intention to treat population for this trial

^e^ 1675 people were enrolled (all with smear‐positive tuberculosis), 105 were later removed for having negative TB cultures and 17 for having MDR TB (as pre‐defined in protocol). 15 were removed for LFTU (“missing” in CONSORT diagram)

^f^ the RAFA trial was a three‐arm trial comparing earlier ART (two weeks) with standard TB treatment versus later ART (eight weeks) with standard TB treatment versus later ART (eight weeks) with high‐dose rifampicin TB treatment. These analyses relate to only 2 out of 3 arms of the trial (leaving out participants assigned to the high‐dose rifampicin arm).

Four trials permitted comparison between starting ART within or at two weeks versus starting ART at between two and eight weeks (the two timepoints in the 2016 WHO recommendation, Comparison A) [25, 30, 31]. The remaining trials used different timepoints and permitted a comparison between ART ≤4 versus >4 weeks (Comparison B). One trial (Amogne *et al*.) randomized people to three arms (ART within one, four and eight weeks) [25]; for Comparison A meta‐analyses, the four‐ and eight‐week groups are combined into “later ART”, whereas for Comparisons B meta‐analyses the one‐ and four‐week groups are combined as “earlier ART” in accordance with our protocol.

**Figure 2:**
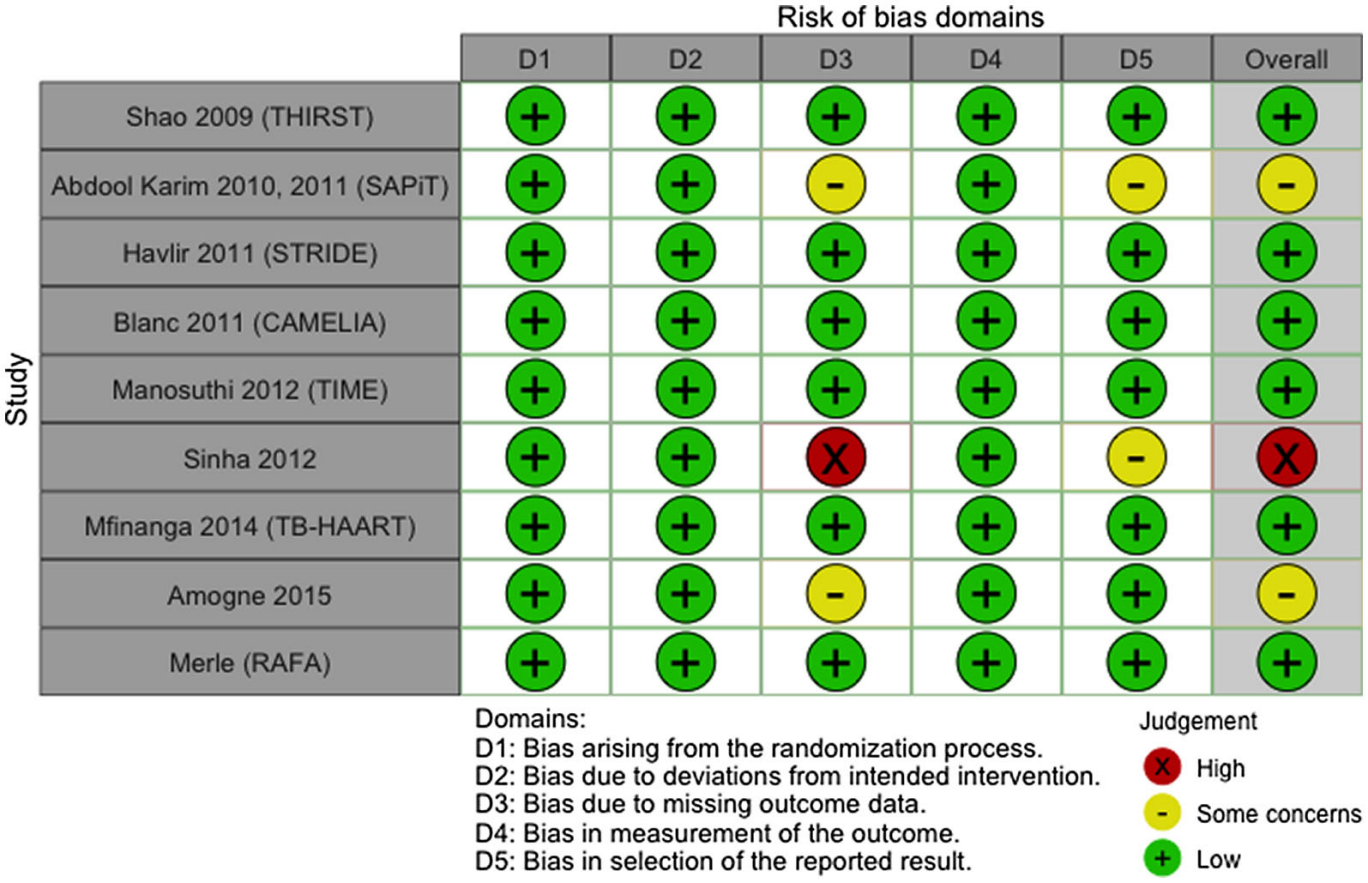
Risk of bias assessment for death outcome (Cochrane ROB2).

Trials had slightly different definitions of endpoints (especially with regards to definitions of serious adverse events), and these are summarized in Table S1. Six included trials were at low risk of bias for the primary outcome of death (Figure 2). We had some concerns of bias for two trials related to high proportions of loss to follow‐up, and high concern of bias in one trial due to very high loss to follow‐up which was different between arms.

### 3.1 Mortality

Overall, weighted mean mortality among all people randomized after one year was 8.6% (Table 2, range 2.9% in TB‐HAART study to 17% in CAMELIA study).

**Table 2:** Death and IRIS outcomes from included studies (other outcomes in Table S2)

Paper	CD4 eligibility	TB type	Earlier ART	Later ART	N people randomized (ITT/mITT)	Deaths by 12 months		IRIS		Sudy reported primary outcome
						Earlier ART group	Later ART group	Earlier ART group	Later ART group	
Shao et al, 2009 (THIRST)	<1200 TLC	Smear pos, any site (except CNS)	2 weeks	8 weeks	70	2/35 (6%)	1/35 (3%)	0/35 (0%)	0/35 (0%)	“TB IRIS were not observed in any subject” “Two deaths and 12 SAEs were observed in early arm versus one death, one clinical failure and seven SAEs in the delayed arm (*p* = 0.60 for time to first grade ¾ event, SAE or death)”
Abdool Karim et al 2010, (SAPiT)^a^	<500	Smear pos PTB	4 and 8 to 12 weeks	26 weeks	642^b^	25/429 (6%)	27/213 (13%)	53/429 (12%)	8/213 (4%)	“There was a reduction in the rate of death among the 429 patients in the combined integrated‐therapy groups (5.4 deaths per 100 person‐years, or 25 deaths), as compared with the 213 patients in the sequential‐therapy group (12.1 per 100 person‐years, or 27 deaths); a relative reduction of 56% (HR, 0.44; 95% CI 0.25 to 0.79; *p* = 0.003).”
Abdool Karim et al 2011, (SAPiT)	<500	Smear pos PTB	4 weeks	8 to 12 weeks	429	15/214 (7%)	15/215 (7%)	43/214 (20%)	18/215 (8%)	“The incidence rate of AIDS or death was 6.9 cases per 100 person‐years in the earlier‐ART group (18 cases) as compared with 7.8 per 100 person‐years in the later‐ART group (19 cases) (incidence‐rate ratio, 0.89; 95% CI, 0.44 to 1.79; *p* = 0.73).”
Havlir et al (STRIDE) 2011	<250	Probable or confirmed, any site.	2 weeks	8 to 12 weeks	806	31/405 (8%)	37/401 (9%)	43/405 (11%)	19/401 (5%)	“In the earlier‐ART group, 12.9% of patients had new AIDS‐defining illness or died by 48 weeks, as compared to 16.1% in the later ART group (95% CI −1.8 to 8.1; *p* = 0.45). Among patients with a screening CD4+ T cells counts of less than 50 per cubic millimeter, 15.5% of patients in the earlier‐ART group versus 26.6% in the later‐ART group had a new AIDS‐defining illness or died (95% CI, 1.5 to 20.5; *p* = 0.02).”
Blanc et al 2011 (CAMELIA)	≤200	Smear pos, any site	2 weeks	8 weeks	661	46/332 (14%)	63/329 (19%)	110/332 (33%)	45/329 (14%)	“The risk of death was significantly reduced in the group that received ART earlier, with 59 deaths among 332 patients (18%) as compared with 90 deaths among 329 patients (27%) in the later ART group (hazard ratio 0.62; 95% confidence interval; 0.44 to 0.86; *p* = 0.006)”^c^
Manosuthi et al. 2012 (TIME)	<350	Probable or confirmed, any site	4 weeks	12 weeks	156	6/79 (8%)	5/77 (6%)	26/79 (33%)	15/77 (19%)	“Eleven (7%) mortalities occurred in a total follow‐up period of 137 patient‐years. Seven percent (6/79, 8.76 per 100 patient‐years) mortalities were in 4‐week group, and 6% (5/77, 7.25 per 100 person‐years) mortalities were in 12‐week group [relative risk 0.845, 95% confidence interval 0.247 to 2.893]”
Sinha et al 2012	Any	Probable or confirmed, any site	2 to 4 weeks	8 to 12 weeks	181	9/92 (10%)	7/89 (8%)	9/92 (10%)	6/89 (7%)	“Kaplan–Meier disease progression‐free survival at 12 months was 79% for early versus 64% for the delayed ART arm (*p* = 0.05)”
Mfinanga et al 2014 TB‐HAART	≥220	Smear and culture‐positive PTB	2 weeks	6 months	1538	19/767 (2%)	21/771 (3%)	81/767 (11%)	93/771 (12%)	“The primary endpoint was reached by 65 (8.5%) of 767 patients in the early ART group versus 71 (9.2%) of 771 in the delayed ART group (relative risk 0.91, CI 0.64 to 1.3; *p* = 0.9)” [NB. Primary endpoint was any of failure of TB treatment, TB recurrence, death within 12 months]
Amogne et al 2015) COMPARISON A^d^	<200	Probable or confirmed, any site (except CNS)	1 week	4 or 8 weeks	478	27/163 (17%)	37/315 (12%)	16/163 (10%)	6/315 (2%)	“All‐cause mortality rates at 48 weeks were 25 per 100 person‐years in week one, 18 per 100 person‐years in week four and 15 per 100 person‐years in week 8 (*p* = 0.2) by the log‐rank test”
Amogne et al 2015 COMPARISON B^d^	<200	Probable or confirmed, any site (except CNS)	1 or 4 weeks	8 weeks	478	47/323 (15%)	17/155 (11%)	22/323 (7%)	0/155 (0%)	
Merle et al (RAFA)	>50	Micro confirmed PTB	2 weeks	8 weeks	498^e^	26/251 (10%)	35/247 (14%)	10/251 (4%)	5/247 (2%)	“There was no evidence that overall mortality differed by treatment arm, with 12 months mortality of 14% [and] 10% in control [and] early ART respectively (*p* = 0.34)”

CNS, central nervous system; PTB, pulmonary TB; Smear pos, sputum smear positive tuberculosis; TLC, total lymphocyte count.

^a^ The 2010 SAPiT report is not included in meta‐analyses as the timepoints used do not match any of our comparisons (i.e. the “earlier ART” group includes people started ≤4 weeks and >4 weeks) and to avoid double counting of participants as these participants also have results reported in the 2011 manuscript

^b^ includes 429 people whose outcomes are also reported in the SAPiT 2011 report

^c^ CAMELIA’s primary outcome was hazard (time‐to‐event) of death measured up until 50 weeks since the final participant was recruited (median follow‐up 25 months per participant). We have extracted data up to 50 weeks per person for our comparison, to increase comparability with other studies

^d^ amogne et al is a study with three arms (one, four, eight weeks). For comparison A (ART ≤2 weeks versus ART >2 weeks ≤8 weeks), the “4 weeks” and “8 weeks” groups are combined. For comparison B (ART ≤4 weeks versus ART >4 weeks), the “1 week” and “4 weeks” groups are combined

^e^ number of people randomly assigned to earlier ART or later ART arm. Not including people assigned to later ART plus high dose rifampicin arm.

**Figure 3:**
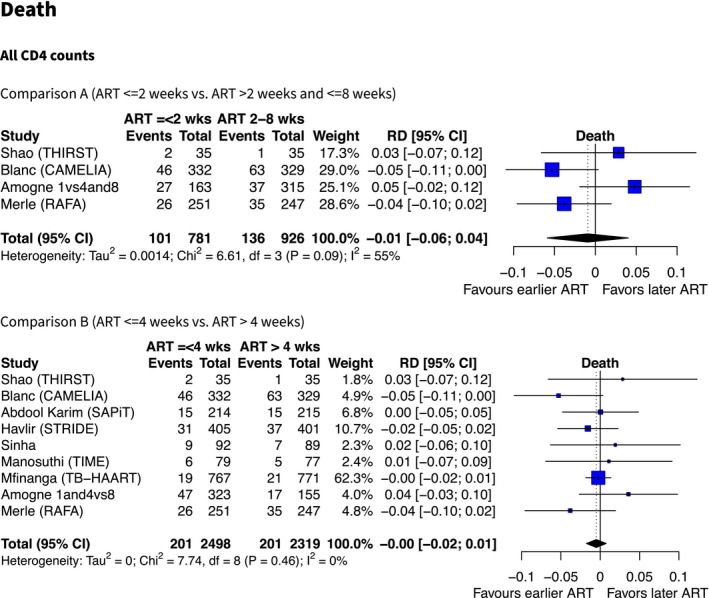
Forest plots showing effect estimates (absolute risk difference) of earlier versus later ART on death (all CD4 counts). Summary estimates are from random effects meta‐regression models, using DerSimonian Laird method to estimate variance.

In the four studies that permitted a comparison of starting ART ≤2 weeks versus two to eight weeks (Comparison A), two studies showed fewer deaths in those starting ART at two weeks (CAMELIA [31] and RAFA, personal communication), whereas two showed more deaths (Amogne *et. al*. [25] and THIRST [30]). All 95% CIs included zero (i.e. null effect). The I^2^ value was 55%, indicating moderate heterogeneity (Figure 3). We estimate an overall effect from our random‐effects model of a risk difference (RD) of −1% (95% CI −6% to +4%).

In nine studies included in Comparison B (≤4 vs. >4 weeks) the absolute risk difference (RD) for mortality for earlier ART (≤4 weeks) compared to later ART (>4 weeks) was 0% (95% CI −2% to +1%, I^2^ = 0%).

Among participants with CD4 counts ≤50 cells/mm^3^, two trials permitted a comparison of starting ART within two weeks versus between two and eight weeks and had data disaggregated by CD4 count. Amogne *et al*. showed a slightly greater proportion of deaths among people starting ART earlier (with a very wide confidence interval, RD +4%, 95% CI −11% to +18%), and CAMELIA showed fewer deaths with earlier ART (−5%, 95% CI −12% to +2%). Meta‐analysis of these two trials gave a combined absolute risk difference of −3% (95% CI −10% to +4%, I^2^ = 8%). Three further trials (STRIDE, SAPiT and TIME) disaggregated data by CD4 cell count strata and used timepoints of four weeks versus eight to twelve or twelve weeks; all of these studies reported fewer deaths in groups with earlier ART in those with low CD4 counts, giving a comparison B (≤4 weeks vs. >4 weeks) RD −6% (five studies, 95% CI −10% to −1%, I^2^ = 0%).

**Figure 4:**
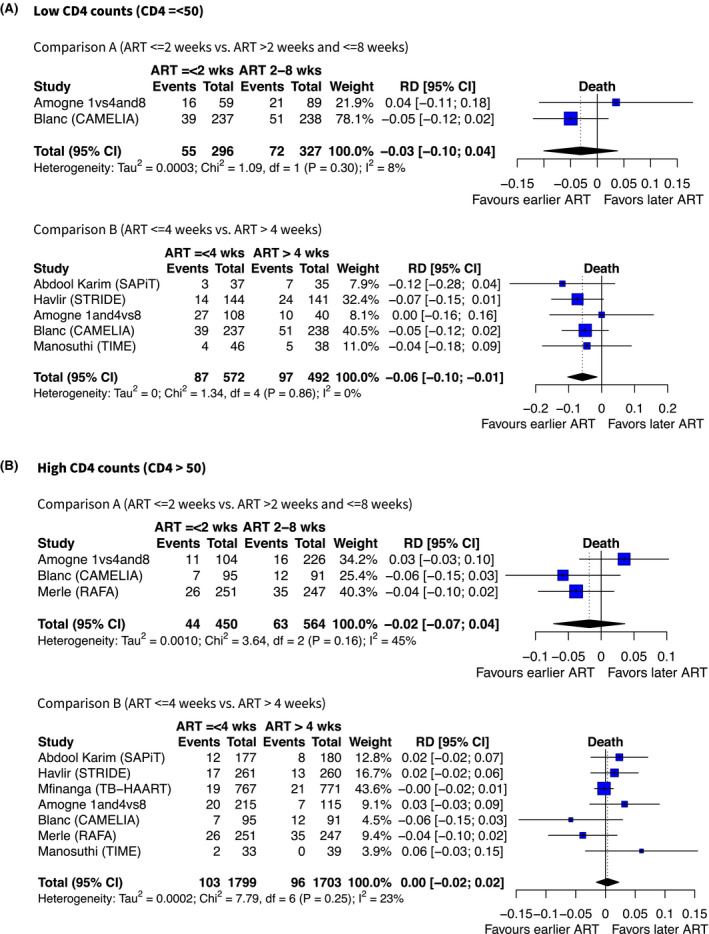
Forest plots showing effect estimates (absolute risk difference) of earlier versus later ART on death (by CD4 count). Summary estimates are from random effects meta‐regression models, using DerSimonian Laird method to estimate variance.

Among participants with higher CD4 counts (>50 cells/mm^3^), Amogne *et al*. found a greater number of deaths in those started on ART within two weeks compared to within eight weeks. The RAFA trial and CAMELIA both showed fewer deaths in the early ART group. Meta‐analysis showed an overall risk difference of −2% (95% CI −7% to +4%, I^2^ = 45%). In the more general comparison of earlier (≤4 weeks) versus later ART (>4 weeks) in people with CD4 cell count >50 cells/mm^3^, there was no difference in mortality by timing of ART, with meta‐estimate of risk difference +1% (95% CI −2% to +3%, I^2^ = 23%), Figure 4.

### 3.2 IRIS

IRIS occurred in 8.5% of participants (weighted mean, range 0% in THIRST trial to 26% in TIME). Most studies assessed IRIS by using definitions defined in Meintjes *et al*. [32]. In the STRIDE and TB‐HAART trials, assessors of IRIS were blinded to ART assignment and taking ART was not a criterion for developing IRIS.

**Figure 5:**
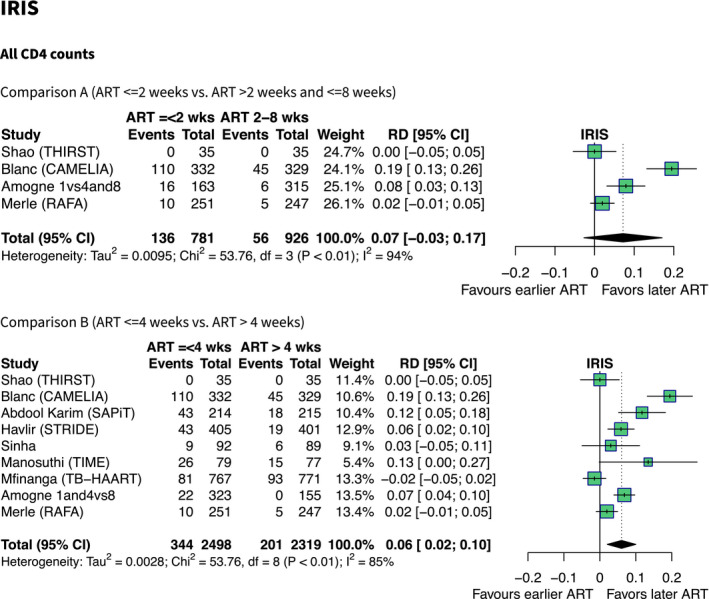
Forest plots showing effect estimates (absolute risk difference) of earlier versus later ART on IRIS (all CD4 counts). Summary estimates are from random effects meta‐regression models, using DerSimonian Laird method to estimate variance.

IRIS was slightly more common among patients who started ART within two weeks compared to between two and eight weeks, but the pooled risk difference failed to reach statistical significance (four studies, RD +7%, 95% CI −3% to +17%, I^2^ = 95%). IRIS was more common among people started on ART ≤4 weeks compared to >4 weeks (nine studies, RD +6%, 95% CI +2% to +10%, I^2^ = 85%) (Figure 5). Among people with CD4 ≤50 cells/mm^3^, IRIS was more common in people started on ART ≤4 weeks compared to >4 weeks, (five studies, +19%, 95% CI +14% to +25%, I^2^ = 36%). Among patients with CD4 > 50 cells/mm^3^, IRIS was slightly more common in early ART groups (seven studies, RD +3%, 0% to +6%, I^2^ = 77%) (Figure in Appendix S3).

### 3.3 AIDS‐defining events

Overall, 8% of all participants (weighted mean) experienced an AIDS‐defining event during study follow‐up (reported in six studies, range 1% in Sinha *et al*. to 15% in TIME). There were slightly fewer AIDS‐defining events in people started on ART ≤2 weeks compared to two to eight weeks (two studies, RD −2%, 95% CI −6% to +3%, I^2^ = 0%) and AIDS‐defining events were slightly less common among people starting ART at ≤4 weeks compared to >4 weeks (six studies, RD −2%, 95% CI −4% to 0%, I^2^ = 0%) — Appendix S3.

In the three studies that reported CD4‐count disaggregated data for AIDS‐defining events (SAPiT, Amogne *et al*. and STRIDE) [25, 26, 29], earlier ART (≤4 weeks) was associated with a reduced risk of AIDS‐defining events in people with CD4 ≤50 cells/mm^3^ (RD −10%, 95% CI −16% to −3%, I^2^ = 0%).

### 3.4 SAEs

There were considerable differences in how SAEs were defined, particularly whether IRIS or AIDS‐defining events were also included as SAEs (see Table S1). One study only reported adverse events related to hepatoxicity [25], six studies reported numbers of SAEs, two reported numbers of people with ≥1 SAE and one study reported both.

Two studies (CAMELIA, TIME) [27, 31] reported treatment‐related SAEs, both counting numbers of events (rather than numbers of people with ≥1 event) and reporting a person‐time denominator, showing similar rates of drug‐related SAEs in earlier versus later ART (Table S2).

### 3.5 HIV viral load suppression

Six studies reported HIV viral load suppression (five studies measured at 12 months, and one study measured at 18 months from randomization). The proportion of participants with viral load results ranged from 24% (RAFA) to 96% (THIRST). There was no difference in the proportion of participants with a suppressed viral load between the earlier ART (≤4 weeks) intervention versus later ART intervention (<4 weeks) (RD 0%, −2% to +3%, I^2^ = 0%) – Appendix S3.

## 4 Discussion

Across nine studies with a variety of timepoints and populations, starting ART earlier (≤4 weeks) compared to later (>4 weeks) had no significant effect on mortality. Among PLHIV with CD4 ≤50 cells/mm^3^, earlier ART reduced mortality. With higher CD4 counts (>50 cells/mm^3^), there was probably no effect of earlier ART on death.

IRIS was slightly more common among people initiating ART early. It is possible that a (probably small) proportion of this apparent effect might be related to reduced mortality in early ART group (as people who have died are no longer at risk of IRIS). A major limitation for IRIS outcome is that only one trial was placebo‐controlled and seven of the nine trials used unblinded outcome assessors, which may lead to bias in non‐mortality outcomes (such as IRIS and AIDS‐defining events). In seven of nine studies, by definition, people not on ART could not develop IRIS, so people in later ART group were “at risk” of IRIS for a shorter time. One of the two studies in which assessors were blinded (TB‐HAART) reported slightly higher rates of IRIS in those starting ART later compared to earlier. The majority of cases of IRIS in all studies were mild or moderate, and IRIS‐related deaths were uncommon (although the cause of death was not ascertained in all studies). Importantly, the increased risk of IRIS was most marked in those with low CD4 cell counts, which is also the group in which there was stronger evidence of overall mortality benefit.

Four studies did not specifically exclude participants with TB meningitis from their trials (although it is not reported how many recruited participants actually had TB meningitis) and separate trial evidence has shown that delayed ART in the setting of TB meningitis is probably beneficial [33], in keeping with other trials showing benefit from delaying ART for cryptococcal meningitis [34]. IRIS is more likely to have serious sequalae in central nervous system TB than in TB at non‐central nervous system sites [18]. There was no significant difference in AIDS‐defining events across the population of people initiating ART, but those with CD4 cell count <50 cells/mm^3^ had a reduced risk of AIDS‐defining events with earlier ART.

Since these trials were conceived and conducted, HIV care in many national programmes has significantly changed. “Treat all” recommendations, coupled with recommendations for rapid and same‐day ART mean that PLHIV without TB start on ART much sooner after diagnosis than would have been the case during the time that most of these trials were being conducted. ART regimens have also changed substantially since these studies; many of these studies used zidovudine‐ or stavudine‐based ART which have higher toxicity and more severe side effect profiles than newer integrase‐inhibitor containing regimens. Integrase inhibitors have been shown to be safe and effective treatments for HIV in people who have TB disease, as demonstrated in two randomized studies (in both of these studies ART was started around six weeks after TB treatment initiation) [35, 36]. None of the included trials in this review used integrase inhibitor containing regimens.

All of these trials were more “explanatory” than “pragmatic” [37, 38], with the possible exception of STRIDE which was conceived as a “strategy” trial [26], resulting in populations which may not be representative of people with TB initiating ART in routine practice. Five of nine trials only included people with microbiologically confirmed TB, whereas in usual practice many people are started on TB treatment for probable TB. All but one trial excluded people who had previously taken and then stopped taking ART and were newly re‐engaged in HIV care. In practice, people restarting ART after a period of not taking ART account for a substantial proportion of ART initiators [39]. Other limitations to the evidence include the lack of data about children and pregnant women (excluded from all trials). Adolescents were included in two trials and data combined with adults. This may limit the generalizability of these conclusions.

Importantly, many of these trials provided intensive, well‐resourced clinical follow‐up as part of study protocols. Trial participants are more likely to be highly motivated and have more frequent monitoring than people with HIV and TB presenting for care in routine national programmes. Randomized trials of earlier ART have shown that, under programmatic conditions, PLHIV without TB who start ART within seven days are more likely to achieve viral suppression and more likely to be retained in care than people who have a longer period between HIV diagnosis and ART initiation [13, 14]. It is not known whether this is the case for PLHIV who also have TB disease. These explanatory trials are not well suited to address how earlier ART for people with TB would impact retention in care under routine service conditions.

None of these trials used steroids to reduce the likelihood and severity of IRIS, a strategy that has been shown to be beneficial in the PredART trial [40] and might be useful to mitigate the effects of IRIS in people starting earlier ART. In PredART, people started ART a median of 16 days after starting TB treatment. Earlier ART initiation may promote better integration between HIV and TB services, and unifying recommendations for people at all CD4 cell counts may also have implementation advantages, particularly given that many programmes no longer require CD4 cell counts before initiating ART. Earlier ART is preferred by patients and is acceptable to at least some national TB programmes (who had already recommended early ART in advance of the March 2021 WHO recommendation). The March 2021 WHO recommendations for earlier ART for everyone are to be welcomed.

As a final limitation, the included trials are clinically heterogeneous in terms of their inclusion criteria (from only people with HIV in hospital to only ambulatory people with higher CD4 counts), their definitions of TB (pulmonary vs. any site and microbiologically proven only versus including clinically diagnosed), and their outcome definitions (especially around IRIS – as discussed above – and severe adverse events). Overall, death proportion varied from 3% to 16%. We used random effects analysis to obtained pooled estimates without assuming the true effect was the same in every trial. Nonetheless, the differences between the included trials – and the differences between all of these trials and the populations of people starting ART with TB in 2021 – mean that some caution should be applied to interpreting these results.

This topic has previously been reviewed in peer‐reviewed literature and to inform earlier WHO guidelines [7, 8]. Since the previous published reviews, data from one new randomized study have been included (RAFA trial, personal communication) and we obtained unpublished CD4‐disaggregated data that were not previously reviewed from two trials [26, 31]. We also have discussed considerations for applying results from these trials (conducted from 2004 to 2014) to the newer conditions in which ART is delivered in 2021 (with integrase inhibitors, “treat all” recommendations, and established guidelines for rapid ART in people without TB). Although considered a key issue for the 2021 ART guidelines, this present question of when to initiate ART in PLHIV who are not already on ART at the time of TB diagnosis may be declining in relevance; concurrent with population increases in ART coverage, HIV‐associated TB is now more commonly diagnosed in people who are already on ART rather than those who are ART naïve. The other important group, but not addressed in this review, are people newly presenting for ART assessment who do not have TB either firmly diagnosed or excluded (i.e. people with TB symptoms who are awaiting a TB test). The SLATE I and SLATE 2 trials indirectly addressed the question of whether to offer same‐day ART concurrent with TB investigations or whether to defer ART to allow the investigation to confirm or refute a TB diagnosis before starting ART.

An individual patient data meta‐analysis using data from all trials may be useful to produce more precise estimates of the effect of earlier compared to later ART, particularly at different CD4 cell strata.

## 5 Conclusion

We identified nine randomized trials comparing earlier ART to later ART in people living with HIV with TB disease who were not taking ART. Most trials were high quality and at low risk of bias for mortality outcomes. The trials tended to be more explanatory than pragmatic, and so may not necessarily reflect how ART is delivered to people with confirmed or probable TB in programmatic settings. Earlier ART (≤4 weeks) reduced mortality at one year in people with CD4 ≤50 cells/mm^3^ (5 trials, risk difference −6%, 95% CI −10% to −1%, I^2^ 0%). In people with higher CD4 counts, there was no evidence of either benefit or harm with earlier ART (≤4 weeks) (seven studies, RD 0%, 95% CI −2% to +2%, I^2^ 23%). IRIS was more common in people starting ART earlier, although IRIS‐related mortality was uncommon. IRIS was more common and increases with earlier ART were most marked in those with CD4 <50, who were also the group with a demonstrated mortality reduction from earlier ART. In addition to evidence from these trials, decisions around the timing of ART initiation should take into account contextual information from more recent observational and trial data (e.g. regarding the safety of integrase inhibitors in TB, improved retention in care from earlier ART in people without TB and reduction in incident IRIS from use of pre‐emptive steroids), as well as service users’ values and preferences, and implementation considerations for HIV programmes. For programmatic logistical and patient preference reasons, earlier ART initiation for everyone with TB and HIV may be preferred to later ART initiation and this recommendation has been included in the March 2021 WHO guidelines.

## Competing interests

AJ and HA are members of the WHO 2021 ART Guideline Development Group. VS is employed by WHO. The findings and conclusions in this paper are those of the authors and do not necessarily represent the official position of the WHO. We declare no financial conflicts of interest and no other conflicts of interest.

## Authors’ contributions

RMB, HMR, RJW, MH and PM designed the systematic review. RMB and HMR screened papers for inclusion and extracted data. RMB, HMR, RJW and PM reviewed included papers. RMB performed statistical meta‐analysis. RMB, HMR and PM wrote the first draft of the manuscript. AJ, HA, MH and RJW revised the manuscript for important intellectual content. All authors contributed to interpreting the data for the work and have read and approved the final version of manuscript.

## Supporting information


**Appendix S1**. Results of search strategy.

**Appendix S2**. PRISMA checklist.

**Table S1**. List of definitons of each of the outcomes for each study
**Table S2**. Table of results for all outcomes

**Figures S1**. Forest plots for all outcomes, for all participants and by CD4 count.

## Data Availability

All data underlying the results are available as part of the article and appendices and no additional source data are required.
