# Supplementary material for: What is the optimum time to start antiretroviral therapy in people with HIV and tuberculosis coinfection? A systematic review and meta‐analysis
Source: J Int AIDS Soc. 2021 Jul 21;24(7):e25772. doi: 10.1002/jia2.25772 (PMC8294654; doi:10.1002/jia2.25772)
Supplement: Supplementary file 1 — Appendix S1. Results of search strategy. [file JIA2-24-e25772-s004.pdf]

Timing or ART in TB: Can antiretroviral therapy in HIV-infected patients with tuberculosis be initiated within 2 weeks than current guidance for ALL CD4 groups?

Search methodology

1 Search methodology 1

1.1 Databases 2

1.2 Clinical trials registers 2

1.3 Information management 2

2 Results 2

3 Appendix: Search strategies 3

3.1 Ovid MEDLINE ALL 3

3.2 Ovid Embase 6

3.3 OvidSP Global Health 9

3.4 Ebsco CINAHL Plus 11

3.5 Ebsco Africa-Wide Information 14

3.6 Wiley Cochrane Central Register of Controlled Trials 16

3.7 Web of Science, Science Citation Index Expanded 18

3.8 Global Index Medicus 19

3.9 clinicaltrials.gov 19

3.10 WHO International Clinical Trials Registry Platform 20

1 Search methodology

A draft search strategy was compiled in the OvidSP Medline database by an experienced information specialist (JF). The search strategy included strings of terms, synonyms and controlled vocabulary terms (where available) to reflect two concepts:

Concept 1: anti-retroviral therapy

Concept 2: tuberculosis

Publication date was limited to 2003 to current and the Cochrane randomised controlled trials filter was added.[1] No other filters or limits were added. Filters and concepts were combined with the Boolean operator AND to find papers which contained both concepts and filters. This search strategy was refined with the project team until the results retrieved reflected the scope of the project. The agreed OvidSP Medline search was adapted for each database to incorporate database-specific syntax and controlled vocabularies. Full details of the search strings used for each database can be found in the appendix.

1.1 Databases

The following bibliographic databases were searched on 12 March 2020.

- Ebsco Africa-Wide Information, complete database
- Ebsco CINAHL Plus, complete database
- Wiley Cochrane Central Register of Controlled Trials, Issue 3 of 12, 2020
- OvidSP Embase, 1974 to 2020 March 11
- OvidSP Global Health, 1910 to 2020 Week 09
- World Health Organization Global Index Medicus, complete database
- OvidSP Medline ALL, 1946 to March 10, 2020
- Clarivate Analytics Web of Science, Science Citation Index Expanded, 1970 to 2020-03-11

1.2 Clinical trials registers

The following clinical trials registers were searched on 12 March 2020:

- Clinical Trials.gov, complete database
- World Health Organization International Clinical Trials Registry Platform, complete database

1.3 Information management

All citations identified by our searches were imported into EndNote X9 software. Duplicates were identified and removed using the method described on the LAS blog.[2]

2 Results

[1] Lefebvre C, Glanville J, Briscoe S, Littlewood A, Marshall C, Metzendorf M-I, Noel-Storr A, Rader T, Shokraneh F, Thomas J, Wieland LS. Technical Supplement to Chapter 4: Searching for and selecting studies. In: Higgins JPT, Thomas J, Chandler J, Cumpston MS, Li T, Page MJ, Welch VA (eds). Cochrane Handbook for Systematic Reviews of Interventions Version 6. Cochrane, 2019, section 3.6. Available from: www.training.cochrane.org/handbook/.

[2] Falconer, Jane, Removing duplicates from an EndNote library. Library & Archives Service Blog: London School of Hygiene & Tropical Medicine. 2018. [online blog] http://blogs.lshtm.ac.uk/library/2018/12/07/removing-duplicates-from-an-endnote-library/.

A total of 4443 results were retrieved by the search. 1975 (44%) were identified as duplicates. Number of results pre-and post-deduplication are listed in the table below.

Database name	Total number of results	Number of results once duplicates removed
Medline	420	395
Embase	1410	1133
Global Health	585	255
CINAHL Plus	246	73
Africa-Wide Information	260	18
Cochrane Central Register of Controlled Trials	609	181
Science Citation Index Expanded	416	101
Global Index Medicus	16	10
International Clinical Trials Registry Platform	346	258
ClinicalTrials.gov	135	44
**Total**	**4443**	**2468**

3 Appendix: Search strategies

This appendix provides full details of all search strings used for bibliographic databases, with dates and number of references returned and notes explaining any unusual search techniques or syntax. The EndNote X9 import order is provided, as the deduplication technique keeps the first uploaded copy of the reference by default.

In all searches, numbers in parentheses at the end of each row show the number of hits retrieved.

3.1 Ovid MEDLINE ALL

Database name	Medline ALL
Database platform	OvidSP
Dates of database coverage	1946 to March 10, 2020
Date searched	12/03/2020
Searched by	JF
Number of results	420

EndNote import order	1 (Medline)<br>3 (Medline in process)
Number of results once duplicates removed	395
Search strategy notes	Search lines ending in a '/' are subject heading searches.<br>Search lines beginning 'exp' are exploded subject heading searches.<br>Two-letter codes at the end of search lines designate the fields to search. Fields codes used are:<br>TI: title<br>AB: abstract<br>KF: author keywords<br>RN: drug registry number/code<br>PT: publication type<br>SH: subject heading<br>or/*x-y* combines search sets in the range *x-y* with Boolean operator OR.<br>* is used for truncation of words.<br>[ ] are used for comments.

1. Antiretroviral Therapy, Highly Active/ (20926)
2. anti-retroviral agents/ (10155)
3. exp anti-hiv agents/ [includes individual drugs] (66472)
4. anti-retroviral*.ti,ab,kf,rn. (13415)
5. antiretroviral*.ti,ab,kf,rn. (61238)
6. HAART.ti,ab,kf,rn. (12033)
7. (ART not "state of the art").ti,ab,kf,rn. (45473)
8. ARV.ti,ab,kf,rn. (3150)
9. ARVs.ti,ab,rf,rn. (1098)
10. zidovudine.ti,ab,kf,rn. (12465)
11. lamivudine.ti,ab,kf,rn. (10082)
12. stavudine.ti,ab,kf,rn. (2981)
13. didanosine.ti,ab,kf,rn. (2808)
14. emtricitabine.ti,ab,kf,rn. (2632)
15. nevirapine.ti,ab,kf,rn. (4628)
16. efavirenz.ti,ab,kf,rn. (4580)
17. tenofovir.ti,ab,kf,rn. (7089)
18. abacavir.ti,ab,kf,rn. (2340)
19. atazanavir.ti,ab,kf,rn. (1806)

20. lopinavir.ti,ab,kf,rn. (2758)
21. ritonavir.ti,ab,kf,rn. (6881)
22. darunavir.ti,ab,kf,rn. (1613)
23. fosamprenavir.ti,ab,kf,rn. (280)
24. indinavir.ti,ab,kf,rn. (2737)
25. saquinavir.ti,ab,kf,rn. (2085)
26. nelfinavir.ti,ab,kf,rn. (1838)
27. tipranavir.ti,ab,kf,rn. (469)
28. trizivir.ti,ab,kf,rn. (69)
29. combivir.ti,ab,kf,rn. (91)
30. kaletra.ti,ab,kf,rn. (161)
31. truvada.ti,ab,kf,rn. (202)
32. duovir.ti,ab,kf,rn. (5)
33. viraday.ti,ab,kf,rn. (1)
34. triomune.ti,ab,kf,rn. (34)
35. odivir.ti,ab,kf,rn. (0)
36. raltegravir.ti,ab,kf,rn. (1887)
37. dolutegravir.ti,ab,kf,rn. (1056)
38. maraviroc.ti,ab,kf,rn. (1121)
39. or/1-38 [ALL ART RESULTS] (147526)
40. exp tuberculosis/ (190121)
41. Mycobacterium tuberculosis/ (49583)
42. tuberculosis.ti,ab,kf. (210993)
43. tuberculin.ti,ab,kf. (15367)
44. tb.ti,ab,kf. (53594)
45. antituberculosis.ti,ab,kf. (5524)
46. att.ti,ab,kf. (3500)
47. or/40-46 [ALL TB RESULTS] (276676)
48. randomized controlled trial.pt. (501347)
49. controlled clinical trial.pt. (93566)
50. randomized.ab. (472443)
51. placebo.ab. (205828)
52. clinical trials as topic.sh. (190322)
53. randomly.ab. (328822)
54. trial.ti. (214726)
55. or/48-54 (1274221)
56. exp animals/ not humans.sh. (4676165)
57. 55 not 56 [COCHRANE RCT FILTER] (1172254)
58. 39 and 47 and 57 (454)
59. limit 58 to yr="2003 -Current" (425)
60. remove duplicates from 59 (420)

3.2 Ovid Embase

Database name	Embase
Database platform	OvidSP
Dates of database coverage	1974 to 2020 March 11
Date searched	12/03/2020
Searched by	JF
Number of results	1410
EndNote import order	2
Number of results once duplicates removed	1133
Search strategy notes	Search lines ending in a '/' are subject heading searches.<br>Search lines beginning 'exp' are exploded subject heading searches.<br>Two-letter codes at the end of search lines designate the fields to search. Fields codes used are:<br>TI: title<br>AB: abstract<br>KW: author keywords<br>DY: drug registry number/code<br>PT: publication type<br>SH: subject heading<br>or/*x*-*y* combines search sets in the range *x*-*y* with Boolean operator OR.<br>* is used for truncation of words.<br>$1 finds words with 0 or 1 character added<br>adj*n* finds terms within *n* words of each other, in any order. If no number is used with adj, the terms are found within 0 terms, in the order entered.<br>[ ] are used for comments

1. exp antiretroviral therapy/ (49690)
2. exp anti human immunodeficiency virus agent/ [includes individual drugs] (156204)

3. anti-retroviral*.ti,ab,kw,dy. (5836)
4. antiretroviral*.ti,ab,kw,dy. (79972)
5. HAART.ti,ab,kw,dy. (17775)
6. (ART not "state of the art").ti,ab,kw,dy. (65341)
7. ARV.ti,ab,kw,dy. (5669)
8. ARVs.ti,ab,kw,dy. (2202)
9. zidovudine.ti,ab,kw,dy. (40429)
10. lamivudine.ti,ab,kw,dy. (38047)
11. stavudine.ti,ab,kw,dy. (15659)
12. didanosine.ti,ab,kw,dy. (14778)
13. emtricitabine.ti,ab,kw,dy. (13343)
14. nevirapine.ti,ab,kw,dy. (18244)
15. efavirenz.ti,ab,kw,dy. (20043)
16. tenofovir.ti,ab,kw,dy. (26600)
17. abacavir.ti,ab,kw,dy. (13090)
18. atazanavir.ti,ab,kw,dy. (8480)
19. lopinavir.ti,ab,kw,dy. (12917)
20. ritonavir.ti,ab,kw,dy. (26814)
21. darunavir.ti,ab,kw,dy. (6193)
22. fosamprenavir.ti,ab,kw,dy. (1523)
23. indinavir.ti,ab,kw,dy. (12595)
24. saquinavir.ti,ab,kw,dy. (10411)
25. nelfinavir.ti,ab,kw,dy. (9927)
26. tipranavir.ti,ab,kw,dy. (2404)
27. trizivir.ti,ab,kw,dy. (73)
28. combivir.ti,ab,kw,dy. (155)
29. kaletra.ti,ab,kw,dy. (251)
30. truvada.ti,ab,kw,dy. (559)
31. duovir.ti,ab,kw,dy. (11)
32. viraday.ti,ab,kw,dy. (2)
33. triomune.ti,ab,kw,dy. (51)
34. odivir.ti,ab,kw,dy. (0)
35. raltegravir.ti,ab,kw,dy. (6268)
36. dolutegravir.ti,ab,kw,dy. (2870)
37. maraviroc.ti,ab,kw,dy. (3955)
38. or/1-37 [ALL ART RESULTS] (258829)
39. exp tuberculosis/ (186365)
40. Mycobacterium tuberculosis/ (61658)
41. tuberculosis.ti,ab,kw. (179852)
42. tuberculin.ti,ab,kw. (13434)
43. tb.ti,ab,kw. (68015)

44. antituberculosis.ti,ab,kw. (6608)
45. att.ti,ab,kw. (4815)
46. or/39-45 [ALL TB RESULTS] (263767)
47. exp randomized controlled trial/ (594223)
48. randomized controlled trial/ (593618)
49. controlled clinical study/ (463565)
50. 48 or 49 (779085)
51. random*.ti,ab. (1510445)
52. randomization/ (86175)
53. intermethod comparison/ (257649)
54. placebo.ti,ab. (302187)
55. (compare or compared or comparison).ti. (502743)
56. ((evaluated or evaluate or evaluating or assessed or assess) and (compare or compared or comparing or comparison)).ab. (2070684)
57. (open adj label).ti,ab. (77401)
58. ((double or single or doubly or singly) adj (blind or blinded or blindly)).ti,ab. (229021)
59. double blind procedure/ (170247)
60. parallel group$1.ti,ab. (25095)
61. (crossover or cross over).ti,ab. (103534)
62. ((assign* or match or matched or allocation) adj5 (alternate or group$1 or intervention$1 or patient$1 or subject$1 or participant$1)).ti,ab. (324077)
63. (assigned or allocated).ti,ab. (381449)
64. (controlled adj7 (study or design or trial)).ti,ab. (341594)
65. (volunteer or volunteers).ti,ab. (243869)
66. human experiment/ (487188)
67. trial.ti. (293023)
68. or/51-67 (4776263)
69. 68 not 50 (4147630)
70. (random* adj sampl* adj7 ("cross section*" or questionnaire$1 or survey* or database$1)).ti,ab. not (comparative study/ or controlled study/ or randomi?ed controlled.ti,ab. or randomly assigned.ti,ab.) (7983)
71. cross-sectional study/ not (randomized controlled trial/ or controlled clinical study/ or controlled study/ or (randomi?ed controlled or control group$1).ti,ab.) (230113)
72. (((case adj control*) and random*) not randomi?ed controlled).ti,ab. (16920)
73. (systematic review not (trial or study)).ti. (137160)
74. (nonrandom* not random*).ti,ab. (15935)
75. "random field*".ti,ab. (2248)
76. (random cluster adj3 sampl*).ti,ab. (1257)
77. (review.ab. and review.pt.) not trial.ti. (783045)
78. "we searched".ab. and (review.ti. or review.pt.) (30914)
79. "update review".ab. (103)

80. (databases adj4 searched).ab. (34076)
81. (rat or rats or mouse or mice or swine or porcine or murine or sheep or lambs or pigs or piglets or rabbit or rabbits or cat or cats or dog or dogs or cattle or bovine or monkey or monkeys or trout or marmoset$1).ti. and animal experiment/ (1049294)
82. Animal experiment/ not (human experiment/ or human/) (2222679)
83. or/70-82 (3414377)
84. 69 not 83 (3631577)
85. 47 or 84 [COCHRANE RCT FILTER] (4225502)
86. 38 and 46 and 85 (1498)
87. limit 86 to yr="2003 -Current" (1453)
88. remove duplicates from 87 (1410)

3.3 OvidSP Global Health

Database name	Global Health
Database platform	OvidSP
Dates of database coverage	1910 to 2020 week 09
Date searched	12/03/2020
Searched by	JF
Number of results	585
EndNote import order	4
Number of results once duplicates removed	255
Search strategy notes	Search lines ending in a '/' are subject heading searches.<br>Search lines beginning 'exp' are exploded subject heading searches.<br>Two-letter codes at the end of search lines designate the fields to search. Fields codes used are:<br>TI: title<br>AB: abstract<br>RN: drug registry number/code<br>or/*x-y* combines search sets in the range *x-y* with Boolean operator OR.

	* is used for truncation of words.<br>$1 finds words with 0 or 1 character added<br>[ ] are used for comments

1. highly active antiretroviral therapy/ (8113)
2. exp antiretroviral agents/ [includes individual drugs] (37112)
3. anti-retroviral*.ti,ab,rn. (1954)
4. antiretroviral*.ti,ab,rn. (37395)
5. HAART.ti,ab,rn. (7437)
6. (ART not "state of the art").ti,ab,rn. (14938)
7. ARV.ti,ab,rn. (1902)
8. ARVs.ti,ab,rn. (723)
9. zidovudine.ti,ab,rn. (4037)
10. lamivudine.ab,rn,ti. (4939)
11. stavudine.ab,rn,ti. (1443)
12. didanosine.ab,rn,ti. (1012)
13. emtricitabine.ab,rn,ti. (1421)
14. nevirapine.ab,rn,ti. (2397)
15. efavirenz.ab,rn,ti. (2312)
16. tenofovir.ab,rn,ti. (3528)
17. abacavir.ab,rn,ti. (1057)
18. atazanavir.ab,rn,ti. (924)
19. lopinavir.ab,rn,ti. (1487)
20. ritonavir.ab,rn,ti. (3241)
21. darunavir.ab,rn,ti. (786)
22. fosamprenavir.ab,rn,ti. (130)
23. indinavir.ab,rn,ti. (985)
24. saquinavir.ab,rn,ti. (687)
25. nelfinavir.ab,rn,ti. (685)
26. tipranavir.ab,rn,ti. (195)
27. trizivir.ab,rn,ti. (15)
28. combivir.ab,rn,ti. (55)
29. kaletra.ab,rn,ti. (40)
30. truvada.ab,rn,ti. (75)
31. duovir.ab,rn,ti. (2)
32. viraday.ab,rn,ti. (0)
33. triomune.ab,rn,ti. (23)
34. odivir.ab,rn,ti. (0)
35. raltegravir.ab,rn,ti. (976)
36. dolutegravir.ab,rn,ti. (475)
37. maraviroc.ab,rn,ti. (461)

38. or/1-37 (57153)
39. exp tuberculosis/ (76410)
40. mycobacterium tuberculosis/ (72223)
41. tuberculosis.ti,ab. (79124)
42. tuberculin.ti,ab. (10388)
43. tb.ti,ab. (26221)
44. antituberculosis.ti,ab. (2136)
45. att.ti,ab. (524)
46. or/39-45 (91062)
47. (rat or rats or mouse or mice or swine or porcine or murine or sheep or lambs or pigs or piglets or rabbit or rabbits or cat or cats or dog or dogs or cattle or bovine or monkey or monkeys or trout or marmoset$1).ti. and animal experiments/ (996)
48. animal experiments/ not man/ (1993)
49. 47 or 48 (2183)
50. randomized controlled trials/ (38624)
51. randomized.ab. (75468)
52. placebo.ab. (35848)
53. randomly.ab. (87962)
54. trial.ab. (85483)
55. groups.ab. (446857)
56. or/50-55 (580274)
57. 56 not 49 (579896)
58. 38 and 46 and 57 (607)
59. limit 58 to yr="2003 -Current" (585)
60. remove duplicates from 59 (585)

3.4 Ebsco CINAHL Plus

Database name	CINAHL Plus
Database platform	Ebsco
Dates of database coverage	Complete database to search date
Date searched	12/03/2020
Searched by	JF
Number of results	246

EndNote import order	5
Number of results once duplicates removed	73
Search strategy notes	Two-letter codes at the beginning of search lines designate the fields to search. Fields codes used are:<br>MH: subject heading<br>TI: title<br>AB: abstract<br>PT: publication type<br>MH terms which end with a + are exploded subject heading terms.<br>* is used for truncation of words.<br>w*n* searches for terms within *n* words of each other.

S1 (MH "Antiretroviral Therapy, Highly Active") (5,960)
S2 (MH "Anti-HIV Agents+") (17,905)
S3 (MH "Anti-Retroviral Agents") (4,881)
S4 (TI anti-retroviral*) OR (AB anti-retroviral*) (742)
S5 (TI antiretroviral*) or (AB antiretroviral*) (16,249)
S6 (TI HAART) OR (AB HAART) (2,298)
S7 (TI ART NOT "state of the art") OR (AB ART NOT "state of the art") (21,318)
S8 (TI ARV) OR (AB ARV) (812)
S9 (TI ARVs) OR (AB ARVs) (792)
S10 (TI zidovudine) OR (AB zidovudine) (833)
S11 (TI lamivudine) OR (AB lamivudine) (1,046)
S12 (TI stavudine) OR (AB stavudine) (292)
S13 (TI didanosine) OR (AB didanosine) (194)
S14 (TI emtricitabine) OR (AB emtricitabine) (643)
S15 (TI nevirapine) OR (AB nevirapine) (710)
S16 (TI efavirenz) OR (AB efavirenz) (850)
S17 (TI tenofovir) OR (AB tenofovir) (1,546)
S18 (TI abacavir) OR (AB abacavir) (401)
S19 (TI atazanavir) OR (AB atazanavir) (398)
S20 (TI lopinavir) OR (AB lopinavir) (507)
S21 (TI ritonavir) OR (AB ritonavir) (1,177)
S22 (TI darunavir) OR (AB darunavir) (391)
S23 (TI fosamprenavir) OR (AB fosamprenavir) (45)
S24 (TI indinavir) OR (AB indinavir) (182)
S25 (TI saquinavir) OR (AB saquinavir) (121)

S26 (TI nelfinavir) OR (AB nelfinavir) (173)
S27 (TI tipranavir) OR (AB tipranavir) (68)
S28 (TI trizivir) OR (AB trizivir) (7)
S29 (TI combivir) OR (AB combivir) (17)
S30 (TI kaletra) OR (AB kaletra) (29)
S31 (TI truvada) OR (AB truvada) (84)
S32 (TI duovir) OR (AB duovir) (0)
S33 (TI viraday) OR (AB viraday) (0)
S34 (TI triomune) OR (AB triomune) (0)
S35 (TI odivir) OR (AB odivir) (0)
S36 (TI raltegravir) OR (AB raltegravir) (426)
S37 (TI dolutegravir) OR (AB dolutegravir) (388)
S38 (TI maraviroc) OR (AB maraviroc) (197)
S39 S1 OR S2 OR S3 OR S4 OR S5 OR S6 OR S7 OR S8 OR S9 OR S10 OR S11 OR S12 OR S13 OR S14 OR S15 OR S16 OR S17 OR S18 OR S19 OR S20 OR S21 OR S22 OR S23 OR S24 OR S25 OR S26 OR S27 OR S28 OR S29 OR S30 OR S31 OR S32 OR S33 OR S34 OR S35 OR S36 OR S37 OR S38 (46,209)
S40 (MH "Tuberculosis+") (21,134)
S41 (MH "Mycobacterium Tuberculosis") (3,615)
S42 (TI tuberculosis) OR (AB tuberculosis) (19,566)
S43 (TI tuberculin) OR (AB tuberculin) (1,282)
S44 (TI tb) OR (AB tb) (9,111)
S45 (TI antituberculosis) OR (AB antituberculosis) (528)
S46 (TI att) OR (AB att) (479)
S47 S40 OR S41 OR S42 OR S43 OR S44 OR S45 OR S46 (28,337)
S48 (MH "Randomized Controlled Trials") (91,146)
S49 (MH "Double-Blind Studies") (43,640)
S50 (MH "Single-Blind Studies") (13,179)
S51 (MH "Random Assignment") (57,780)
S52 (MH "Pretest-Posttest Design") (39,789)
S53 (MH "Cluster Sample") (4,069)
S54 (TI randomised OR randomized) (228,335)
S55 (AB random*) (283,847)
S56 (TI trial) (100,125)
S57 (MH "sample size") AND (AB assigned OR allocated OR control) (5,851)
S58 (MH placebos) (11,653)
S59 (PT "randomized controlled trial") (86,220)
S60 (AB control w5 group) (100,124)
S61 (MH "crossover design") OR (MH "comparative studies") (259,379)
S62 (AB cluster w3 rct) (327)
S63 (MH "Animals+") (87,843)

S64 (MH "animal studies") (111,306)
S65 (TI "animal model*") (2,719)
S66 S63 OR S64 OR S65 (191,272)
S67 (MH human) (2,044,546)
S68 S66 NOT S67 (168,231)
S69 S48 OR S49 OR S50 OR S51 OR S52 OR S53 OR S54 OR S55 OR S56 OR S57 OR S58 OR S59 OR S60 OR S61 OR S62 (663,913)
S70 S69 NOT S68 (635,119)
S71 S39 AND S47 AND S70 (252)
S72 S71 Limiters - Published Date: 20030101-20201231 (246)

3.5 Ebsco Africa-Wide Information

Database name	Africa-Wide Information
Database platform	Ebsco
Dates of database coverage	Complete database to search date
Date searched	12/03/2020
Searched by	JF
Number of results	260
EndNote import order	6
Number of results once duplicates removed	18
Search strategy notes	Two-letter codes at the beginning of search lines designate the fields to search. Fields codes used are:<br>MH: subject heading<br>TI: title<br>AB: abstract<br>PT: publication type<br>MH terms which end with a + are exploded subject heading terms.<br>* is used for truncation of words.<br>w*n* searches for terms within *n* words of each other.

S1 (TI anti-retroviral*) OR (AB anti-retroviral*) (1,527)

S2 (TI antiretroviral*) or (AB antiretroviral*) (17,107)

S3 (TI HAART) OR (AB HAART) (2,765)

S4 (TI ART NOT "state of the art") OR (AB ART NOT "state of the art") (32,433)

S5 (TI ARV) OR (AB ARV) (1,804)

S6 (TI ARVs) OR (AB ARVs) (1,174)

S7 (TI zidovudine) OR (AB zidovudine) (1,279)

S8 (TI lamivudine) OR (AB lamivudine) (912)

S9 (TI stavudine) OR (AB stavudine) (588)

S10 (TI didanosine) OR (AB didanosine) (214)

S11 (TI emtricitabine) OR (AB emtricitabine) (295)

S12 (TI nevirapine) OR (AB nevirapine) (1,626)

S13 (TI efavirenz) OR (AB efavirenz) (1,014)

S14 (TI tenofovir) OR (AB tenofovir) (917)

S15 (TI abacavir) OR (AB abacavir) (227)

S16 (TI atazanavir) OR (AB atazanavir) (212)

S17 (TI lopinavir) OR (AB lopinavir) (482)

S18 (TI ritonavir) OR (AB ritonavir) (772)

S19 (TI darunavir) OR (AB darunavir) (178)

S20 (TI fosamprenavir) OR (AB fosamprenavir) (29)

S21 (TI indinavir) OR (AB indinavir) (168)

S22 (TI saquinavir) OR (AB saquinavir) (118)

S23 (TI nelfinavir) OR (AB nelfinavir) (131)

S24 (TI tipranavir) OR (AB tipranavir) (37)

S25 (TI trizivir) OR (AB trizivir) (9)

S26 (TI combivir) OR (AB combivir) (26)

S27 (TI kaletra) OR (AB kaletra) (13)

S28 (TI truvada) OR (AB truvada) (65)

S29 (TI duovir) OR (AB duovir) (1)

S30 (TI viraday) OR (AB viraday) (0)

S31 (TI triomune) OR (AB triomune) (36)

S32 (TI odivir) OR (AB odivir) (0)

S33 (TI raltegravir) OR (AB raltegravir) (202)

S34 (TI dolutegravir) OR (AB dolutegravir) (234)

S35 (TI maraviroc) OR (AB maraviroc) (71)

S36 S1 OR S2 OR S3 OR S4 OR S5 OR S6 OR S7 OR S8 OR S9 OR S10 OR S11 OR S12 OR S13 OR S14 OR S15 OR S16 OR S17 OR S18 OR S19 OR S20 OR S21 OR S22 OR S23 OR S24 OR S25 OR S26 OR S27 OR S28 OR S29 OR S30 OR S31 OR S32 OR S33 OR S34 OR S35 (48,435)

S37 (TI tuberculosis) OR (AB tuberculosis) (175,811)

S38 (TI tuberculin) OR (AB tuberculin) (7,543)

S39 (TI tb) OR (AB tb) (31,357)
S40 (TI antituberculosis) OR (AB antituberculosis) (3,794)
S41 (TI att) OR (AB att) (549)
S42 S37 OR S38 OR S39 OR S40 OR S41 (180,105)
S43 S36 AND S42 (3,637)
S44 (AB randomized) (16,996)
S45 (AB placebo) (6,480)
S46 (AB randomly) (21,374)
S47 (TI trial) (19,874)
S49 S44 OR S45 OR S46 OR S47 (53,761)
S50 S43 AND S49 (280)
S51 S50 Limiters - Year Published: 2003-2020 (260)

3.6 Wiley Cochrane Central Register of Controlled Trials

Database name	Cochrane Central Register of Controlled Trials
Database platform	Wiley
Dates of database coverage	Issue 3 of 12, March 2020
Date searched	12/03/2020
Searched by	JF
Number of results	609
EndNote import order	8
Number of results once duplicates removed	181
Search strategy notes	* is used for truncation.<br>Searches ending :ti,ab,kw search the title, abstract and keywords.<br>The Cochrane Randomised Controlled Trial filter is not used here, as all results should be RCTs.

#1 MeSH descriptor: [Antiretroviral Therapy, Highly Active] this term only (1168)
#2 MeSH descriptor: [Anti-Retroviral Agents] this term only (647)
#3 MeSH descriptor: [Anti-HIV Agents] explode all trees (3361)

#4 (anti-retroviral*):ti,ab,kw (1007)
#5 (antiretroviral*):ti,ab,kw (8744)
#6 (HAART):ti,ab,kw (1278)
#7 (ART NOT "state of the art"):ti,ab,kw (6531)
#8 (ARV):ti,ab,kw (655)
#9 (ARVs):ti,ab,kw (213)
#10 (zidovudine):ti,ab,kw (2122)
#11 (lamivudine):ti,ab,kw (3077)
#12 (stavudine):ti,ab,kw (726)
#13 (didanosine):ti,ab,kw (696)
#14 (emtricitabine):ti,ab,kw (1785)
#15 (nevirapine):ti,ab,kw (1005)
#16 (efavirenz):ti,ab,kw (1501)
#17 (tenofovir):ti,ab,kw (3166)
#18 (abacavir):ti,ab,kw (830)
#19 (atazanavir):ti,ab,kw (732)
#20 (lopinavir):ti,ab,kw (1134)
#21 (ritonavir):ti,ab,kw (2784)
#22 (darunavir):ti,ab,kw (637)
#23 (fosamprenavir):ti,ab,kw (116)
#24 (indinavir):ti,ab,kw (383)
#25 (saquinavir):ti,ab,kw (345)
#26 (nelfinavir):ti,ab,kw (348)
#27 (tipranavir):ti,ab,kw (119)
#28 (trizivir):ti,ab,kw (35)
#29 (combivir):ti,ab,kw (81)
#30 (kaletra):ti,ab,kw (141)
#31 (truvada):ti,ab,kw (229)
#32 (duovir):ti,ab,kw (1)
#33 (viraday):ti,ab,kw (0)
#34 (triomune):ti,ab,kw (12)
#35 (odivir):ti,ab,kw (0)
#36 (raltegravir):ti,ab,kw (668)
#37 (dolutegravir):ti,ab,kw (438)
#38 (maraviroc):ti,ab,kw (347)
#39 #1 or #2 or #3 or #4 or #5 or #6 or #7 or #8 or #9 or #10 or #11 or #12 or #13 or #14 or #15 or #16 or #17 or #18 or #19 or #20 or #21 or #22 or #23 or #24 or #25 or #26 or #27 or #28 or #29 or #30 or #31 or #32 or #33 or #34 or #35 or #36 or #37 or #38 (18315)
#40 MeSH descriptor: [Tuberculosis] explode all trees (557)
#41 MeSH descriptor: [Mycobacterium tuberculosis] this term only (302)

#42 (tuberculosis):ti,ab,kw (6216)
#43 (tuberculin):ti,ab,kw (738)
#44 (tb):ti,ab,kw (3041)
#45 (antituberculosis):ti,ab,kw (498)
#46 (att):ti,ab,kw (284)
#47 #40 or #41 or #42 or #43 or #45 or #46 (6755)
#48 #39 and #47 (640)
#49 #48 with Publication Year from 2003 to 2020, in Trials (609)

3.7 Web of Science, Science Citation Index Expanded

Database name	Science Citation Index Expanded
Database platform	Clarivate Analytics Web of Science
Dates of database coverage	1970 – 11/03/2020
Date searched	12/03/2020
Searched by	JF
Number of results	416
EndNote import order	7
Number of results once duplicates removed	101
Search strategy notes	TOPIC searches search in the title, abstract and keywords fields.<br>AB searches search in the abstract<br>TI searches search in the title<br>* is used for truncation of words.<br>All searches run on Timespan=2003-2020.

#1 TOPIC: (anti-retroviral* or antiretroviral* or HAART or (ART NOT "state of the art") or ARV or ARVs or zidovudine or lamivudine or stavudine or didanosine or emtricitabine or nevirapine or efavirenz or tenofovir or abacavir or atazanavir or lopinavir or ritonavir or darunavir or fosamprenavir or indinavir or saquinavir or nelfinavir or tipranavir or trizivir or combivir or kaletra or truvada or duovir or viraday or triomune or odivir or raltegravir or dolutegravir or maraviroc) (143,425)

#2 TOPIC: (tuberculosis or tuberculin or tb or antituberculosis or att) (130,135)
#3 AB=(randomized or placebo or randomly) (608,632)
#4 TI=(trial) (286,011)
#5 #3 OR #4 (786,904)
#6 #1 AND #2 AND #5 (416)

3.8 Global Index Medicus

Database name	Global Index Medicus
Database platform	World Health Organization
Dates of database coverage	Complete database to date of search
Date searched	12/03/2020
Searched by	JF
Number of results	16
EndNote import order	9
Number of results once duplicates removed	10
Search strategy notes	TOPIC searches search in the title, abstract and keywords fields.<br>AB searches search in the abstract<br>TI searches search in the title<br>* is used for truncation of words.<br>All searches run on Timespan=2003-2020.

(anti-retroviral* OR antiretroviral* OR haart OR (art not "state of the art") OR arv OR arvs OR zidovudine OR lamivudine OR stavudine OR didanosine OR emtricitabine OR nevirapine OR efavirenz OR tenofovir OR abacavir OR atazanavir OR lopinavir OR ritonavir OR darunavir OR fosamprenavir OR indinavir OR saquinavir OR nelfinavir OR tipranavir OR trizivir OR combivir OR kaletra OR truvada OR duovir OR viraday OR triomune OR odivir OR raltegravir OR dolutegravir OR maraviroc) AND (tuberculosis OR tuberculin OR tb OR antituberculosis OR att) AND (randomized OR placebo OR randomly OR trial) AND (year_cluster:[2003 TO 2020])

3.9 clinicaltrials.gov

Database name	clinicaltrials.gov
Database platform	-
Dates of database coverage	Complete database to search date
Date searched	12/03/2020
Searched by	JF
Number of results	97: search 1<br>38: search 2
EndNote import order	11
Number of results once duplicates removed	44
Search strategy notes	The following search was completed on the Advanced Search page. The description provides field name then search terms. All other fields were left as default.

Search 1
Condition or disease: tuberculosis OR tb
Intervention/treatment: anti-retroviral* OR antiretroviral* OR HAART OR ART OR ARV OR ARVs OR zidovudine OR lamivudine OR stavudine OR didanosine OR emtricitabine OR nevirapine OR efavirenz OR tenofovir OR abacavir OR atazanavir OR lopinavir

Search 2
Condition or disease: tuberculosis OR tb
Intervention/treatment: ritonavir OR darunavir OR fosamprenavir OR indinavir OR saquinavir OR nelfinavir OR tipranavir OR raltegravir OR dolutegravir OR maraviroc

3.10 WHO International Clinical Trials Registry Platform

Database name	International Clinical Trials Registry Platform
Database platform	http://apps.who.int/trialsearch/AdvSearch.aspx
Dates of database coverage	Complete database to search date

Date searched	12/03/2020
Searched by	JF
Number of results	260: search 1<br>28: search 2<br>58: search 3
EndNote import order	10
Number of results once duplicates removed	258
Search strategy notes	Searches run on the advanced search page.

Search 1
Condition: tuberculosis OR TB
Intervention: anti-retroviral* or antiretroviral* or HAART or ART or ARV or ARVs

Search 2
Condition: tuberculosis OR TB
Intervention: zidovudine or lamivudine or stavudine or didanosine or emtricitabine or nevirapine or efavirenz or tenofovir or abacavir

Search 3
Condition: tuberculosis OR TB
Intervention: atazanavir or lopinavir or ritonavir or darunavir or indinavir or saquinavir or nelfinavir or raltegravir or dolutegravir or maraviroc
