# Supplementary material for: What is the optimum time to start antiretroviral therapy in people with HIV and tuberculosis coinfection? A systematic review and meta‐analysis
Source: J Int AIDS Soc. 2021 Jul 21;24(7):e25772. doi: 10.1002/jia2.25772 (PMC8294654; doi:10.1002/jia2.25772)
Supplement: Supplementary file 2 — Appendix S2. PRISMA checklist. [file JIA2-24-e25772-s001.pdf]

PRISMA 2009 Checklist

Section/topic	#	Checklist item	Reported on page #
**TITLE**			
Title	1	**Identify the report as a systematic review, meta-analysis, or both.**<br><br>“What is the optimum time to start antiretroviral therapy in people with HIV and tuberculosis coinfection? A systematic review and meta-analysis.”	Yes (page 1)
**ABSTRACT**			
Structured summary	2	**Provide a structured summary including, as applicable: background; objectives; data sources; study eligibility criteria, participants, and interventions; study appraisal and synthesis methods; results; limitations; conclusions and implications of key findings; systematic review registration number.**<br><br>See paper abstract	Abstract
**INTRODUCTION**			
Rationale	3	**Describe the rationale for the review in the context of what is already known.**<br><br>“The 2016 WHO recommendations for people with CD4 < 50 to start ART within two weeks and others to start ART within eight weeks may introduce logistical complexity, particularly in situations where CD4 cell counts are not readily available. Furthermore, rapid ART (within seven days) is now recommended for most PLHIV without TB, as evidence suggests that reducing delay between diagnosis of HIV and starting ART improves outcomes. [6,7] It is unknown whether this is also the case in people coinfected with TB and HIV. …. In light of the still unacceptably high levels of mortality associated with HIV and TB coinfection, and the trend towards earlier initiation of ART in PLHIV without TB, we sought to review the evidence around the timing of ART initiation PLHIV who have TB disease.”	Page 3
Objectives	4	**Provide an explicit statement of questions being addressed with reference to participants, interventions, comparisons, outcomes, and study design (PICOS).**<br><br>“We included studies of PLHIV of any age, in any country setting. We included two sets of interventions and comparators. Firstly, we compared starting ART within two weeks to starting ART between two and eight weeks	Page 4

		after TB treatment as these are the two strategies recommended in 2016 WHO guidance (**Comparison A**). However, we included a comparison with a more general definition (**Comparison B**) because some trials used different time intervals to define early and delayed ART initiation (e.g. compared four weeks to twelve weeks). We analysed data across all CD4 cell counts, and by CD4 cell count strata using a cut-point of CD4 count 50 cells/mm^3 to reflect current WHO guidelines. These categories were pre-specified. The main outcome of interest was mortality; secondary outcomes included incidence of IRIS, AIDS defining events, serious adverse events (SAEs), viral load suppression and loss to follow-up.”	
**METHODS**			
Protocol and registration	5	**Indicate if a review protocol exists, if and where it can be accessed (e.g., Web address), and, if available, provide registration information including registration number.**<br><br>The protocol and search strategy are available online at PROSPERO (CRD42020190396).	Page 4
Eligibility criteria	6	**Specify study characteristics (e.g., PICOS, length of follow-up) and report characteristics (e.g., years considered, language, publication status) used as criteria for eligibility, giving rationale.**<br><br>“We included studies of PLHIV of any age, in any country setting. We included two sets of interventions and comparators. Firstly, we compared starting ART within two weeks to starting ART between two and eight weeks after TB treatment as these are the two strategies recommended in 2016 WHO guidance (**Comparison A**). However, we included a comparison with a more general definition (**Comparison B**) because some trials used different time intervals to define early and delayed ART initiation (e.g. compared four weeks to twelve weeks). We analysed data across all CD4 cell counts, and by CD4 cell count strata using a cut-point of CD4 count 50 cells/mm^3 to reflect current WHO guidelines. These categories were pre-specified. The main outcome of interest was mortality; secondary outcomes included incidence of IRIS, AIDS defining events, serious adverse events (SAEs), viral load suppression and loss to follow-up.”	Page 4
Information sources	7	**Describe all information sources (e.g., databases with dates of coverage, contact with study authors to identify additional studies) in the search and date last searched.**	Page 4 and

		We used a broad search strategy to identify randomised controlled trials (RCTs) that compared timing of ART in people with HIV and TB. The search was designed with the assistance of a specialist librarian. We searched eight databases including Medline and Embase and two clinical trial registries on 12 March 2020 (Appendix 1).	appendix 1
Search	8	**Present full electronic search strategy for at least one database, including any limits used, such that it could be repeated.**	Appendix 1
Study selection	9	**State the process for selecting studies (i.e., screening, eligibility, included in systematic review, and, if applicable, included in the meta-analysis).**<br><br>All titles and abstracts were reviewed by RMB and HMR using the Rayaan software program.[14] After reviewing an initial 10% sample of titles/abstracts in duplicate, and determining that there was 100% agreement between reviewers, the remainder of title abstracts were reviewed by one reviewer only. All papers at full text review stage were reviewed by both RMB and HMR for decision on inclusion. Both RMB and HMR extracted data independently in duplicate from studies selected for inclusion	Page 4
Data collection process	10	**Describe method of data extraction from reports (e.g., piloted forms, independently, in duplicate) and any processes for obtaining and confirming data from investigators.**<br><br>Both RMB and HMR extracted data independently in duplicate from studies selected for inclusion. Data were extracted from published manuscripts, supplementary data files and study protocols and entered into a spreadsheet.	Page 4
Data items	11	**List and define all variables for which data were sought (e.g., PICOS, funding sources) and any assumptions and simplifications made.**<br>Appendix 3 (supplementary table 1 lists definitions for each study of each variable)	Appendix 3
Risk of bias in individual studies	12	**Describe methods used for assessing risk of bias of individual studies (including specification of whether this was done at the study or outcome level), and how this information is to be used in any data synthesis.**<br><br>We used the Cochrane Risk of Bias (ROB) 2 tool [19] to assess study quality. Risk of bias assessments were performed in duplicate by HMR and RMB and differences resolved by consensus and discussion with PM.	Page 5
Summary measures	13	**State the principal summary measures (e.g., risk ratio, difference in means).**	Page 5

		We expressed effects as absolute risk differences	
Synthesis of results	14	**Describe the methods of handling data and combining results of studies, if done, including measures of consistency (e.g., I^2) for each meta-analysis.**<br><br>Data were pooled in meta-analysis using package "meta" [15] in R.[16] We expressed effects as absolute risk differences. Random effects meta-analysis was used because we anticipated heterogeneity in results between studies. We used the Mantel-Haenszel method to estimate confidence intervals of risk differences, and the DerSimonian Laird method to estimate variance of pooled effect.[17]	Page 5

Section/topic	#	Checklist item	Reported on page #
Risk of bias across studies	15	**Specify any assessment of risk of bias that may affect the cumulative evidence (e.g., publication bias, selective reporting within studies).**<br>A major limitation for IRIS outcome is that only one trial was placebo-controlled and seven of the nine trials used unblinded outcome assessors, which may lead to bias in non-mortality outcomes (such as IRIS and AIDS defining events).	Page 20
Additional analyses	16	**Describe methods of additional analyses (e.g., sensitivity or subgroup analyses, meta-regression), if done, indicating which were pre-specified.**<br><br>We analysed data across all CD4 cell counts, and by CD4 cell count strata using a cut-point of CD4 count 50 cells/mm^3 to reflect current WHO guidelines. These categories were pre-specified.	Page 5
**RESULTS**			
Study selection	17	**Give numbers of studies screened, assessed for eligibility, and included in the review, with reasons for exclusions at each stage, ideally with a flow diagram.**<br><br>PRIMSA diagram is figure 1	Page 6
Study characteristics	18	**For each study, present characteristics for which data were extracted (e.g., study size, PICOS, follow-up period) and provide the citations.**<br><br>Table 1 and table 2	Page 8-13
Risk of bias within studies	19	**Present data on risk of bias of each study and, if available, any outcome level assessment (see item 12).**<br><br>Figure 2	Page 14

Results of individual studies	20	**For all outcomes considered (benefits or harms), present, for each study: (a) simple summary data for each intervention group (b) effect estimates and confidence intervals, ideally with a forest plot.**<br><br>Figures 3,4,5 and appendix 3	Figures 3,4,5 and appendix 3
Synthesis of results	21	**Present results of each meta-analysis done, including confidence intervals and measures of consistency.**<br><br>Figures 3,4,5 and appendix 3	Figures 3,4,5 and appendix 3
Risk of bias across studies	22	**Present results of any assessment of risk of bias across studies (see Item 15).**<br><br>A major limitation for IRIS outcome is that only one trial was placebo-controlled and seven of the nine trials used unblinded outcome assessors, which may lead to bias in non-mortality outcomes (such as IRIS and AIDS defining events). In seven of nine studies, by definition, people not on ART couldn't develop IRIS, so people in later ART group were 'at risk' of IRIS for a shorter time	Page 20
Additional analysis	23	**Give results of additional analyses, if done (e.g., sensitivity or subgroup analyses, meta-regression [see Item 16]).**<br>Appendix 3	Appendix 3
**DISCUSSION**			
Summary of evidence	24	**Summarize the main findings including the strength of evidence for each main outcome; consider their relevance to key groups (e.g., healthcare providers, users, and policy makers).**<br><br>Across nine studies with a variety of timepoints and populations, starting ART earlier (≤ 4 weeks) compared to later (>4 weeks) had no significant effect on mortality (absolute risk difference -0%, 95% CI -2% to +1%). Among PLHIV with CD4 ≤50 cells/mm^3, earlier ART was associated with reduced mortality (5 studies, RD -6%, 95% CI -10% to -1%). With higher CD4 counts (> 50 cells/mm^3), there was probably no effect of earlier ART on death (RD 0%, 95% CI -2% to +2%).	Page 20
Limitations	25	**Discuss limitations at study and outcome level (e.g., risk of bias), and at review-level (e.g., incomplete retrieval of identified research, reporting bias).**<br><br>ART regimens have also changed substantially since these studies; many of these studies used zidovudine- or stavudine- based ART which have higher toxicity and more severe side effect profiles than newer integrase-inhibitor	Page 21

		containing regimens. Integrase inhibitors have been shown to be safe and effective treatments for HIV in people who have TB disease. [31,32] None of these trials used integrase inhibitor containing regimens.<br><br>All of these trials were more "explanatory" than "pragmatic",[33,34] with the possible exception of STRIDE which was conceived as a 'strategy' trial[21], resulting in populations which may not be representative of people with TB initiating ART in routine practice. Five of nine trials only included people with microbiologically-confirmed TB whereas in usual practice many people are started on TB treatment for probable TB. All but one trial excluded people who had previously taken and then stopped taking ART and were newly re-engaged in HIV care. In practice, people restarting ART after a period of not taking ART account for a substantial proportion of ART initiators. [35] Other limitations to the evidence include the lack of data about children and pregnant women (excluded from all trials). Adolescents were included in two trials and data combined with adults. This may limit the generalisability of these conclusions.	
Conclusions	26	**Provide a general interpretation of the results in the context of other evidence, and implications for future research.**<br><br>For programmatic logistical and patient preference reasons, earlier ART initiation for everyone with TB and HIV may be preferred to later ART.	Page 22
**FUNDING**			
Funding	27	**Describe sources of funding for the systematic review and other support (e.g., supply of data); role of funders for the systematic review.**<br><br>This work was funded in part by WHO Global HIV, Hepatitis and STI programme, who commissioned this review in order to inform the 2021 ART Guidelines. An independent methodologist contracted to WHO provided some technical expertise about review methods. The findings and conclusions in this paper are those of the authors and do not necessarily represent the official position of the WHO. RMB and PM are funded by Wellcome; grant reference numbers 203905/Z/16/Z and 206575/Z/17/Z, respectively. RJW is funded by Wellcome (104803, 203133) and also receives support from Francis Crick Insitute, which is funded by UKRI, CRUK and Wellcome. Neither	Page 5

		Wellcome, UKRI nor CRUK had any role in the design or analysis of this systematic review.	

From: Moher D, Liberati A, Tetzlaff J, Altman DG, The PRISMA Group (2009). Preferred Reporting Items for Systematic Reviews and Meta-Analyses: The PRISMA Statement. PLoS Med 6(7): e1000097. doi:10.1371/journal.pmed1000097

For more information, visit: **www.prisma-statement.org**.
