# Supplementary material for: What is the optimum time to start antiretroviral therapy in people with HIV and tuberculosis coinfection? A systematic review and meta‐analysis
Source: J Int AIDS Soc. 2021 Jul 21;24(7):e25772. doi: 10.1002/jia2.25772 (PMC8294654; doi:10.1002/jia2.25772)
Supplement: Supplementary file 3 — Table S1. List of definitons of each of the outcomes for each study Table S2. Table of results for all outcomes [file JIA2-24-e25772-s002.pdf]

**Supplementary table 1:** Definitions of outcomes from each trial

| | Death | AIDS-defining events | Immune Reconstitution Inflammatory Syndrome | Serious adverse events | Loss to follow-up | Viral load suppression | ART switch |
|---|---|---|---|---|---|---|---|
| Shao et al, 2009 (THIRST) | All-cause mortality at one year (1) | Not specifically defined (2) | "a) new persistent fevers (temperature >101.5ºF) developing after the initiation of ART, and not believed to be associated with ART and without an identifiable source, b) marked worsening or emergence of intrathoracic lymphadenopathy, pulmonary infiltrates or pleural effusions on radiologic examination, or c) worsening or emergence of lymphadenopathy on serial examinations or worsening of other tuberculous lesions." Up to week 104. | "Any untoward medical occurrence that resulted in death, was considered life-threatening, required inpatient hospitalization or prolongation of existing hospitalization beyond what was required in the study, or resulted in persistent or resulted in significant disability/incapacity." Up to week 104. Number of events (not number of participants with ≥1 event). Includes AIDS-defining events. | Not specifically defined, outcomes of all participants ascertained at week 104. | HIV-1 RNA <400 copies/ml at 48 weeks (3) | Not specifically defined, reported up to week 48 |
| Abdool Karim et al 2010, 2011 (SAPiT) | All-cause mortality at 18 months | "AIDS-defining illness" | All cases of IRIS identified during the trial were retrospectively assessed and were found to meet the 2008 IRIS definition of one major or two minor clinical criteria (Meintjes et al) [27] | Grade 3-4 non-IRIS adverse events by 18 months. Number of participants with ≥1 event reported in main paper. Includes AIDS-defining illnesses. | No visit within 4 months or requested withdrawal, relocated or were unable to comply with study protocol | HIV-1 RNA <400 copies/mL at 12 months | NA |
| Havlir et al (STRIDE) 2011 | All-cause mortality by 48 weeks | "An independent reviewer who was unaware of the study group assignment assessed new AIDS-defining events on the basis of the standardized definitions of the AIDS Clinical Trail Group." | "A reviewer who was unaware of the study-group assignments confirmed tuberculosis-associated IRIS cases on the basis of at least one major or two minor clinical criteria (Meintjes et al 2008). [27] Concurrent ART was not required for a patient to be classified as having tuberculosis-associated IRIS." | "Adverse events were graded with the use of the DAIDS Table for Grading the Severity of Adult and Paediatric Adverse events". Up to 48 weeks. Number of participants with ≥1 event. | Not specifically defined, reported up to 48 weeks. | HIV-1 RNA <400 copies/mL at 48 weeks | Not specifically defined, reported up to 48 weeks. |
| Blanc et al 2011 | All-cause mortality | NA | "Worsening or emergence of TB symptoms after the initiation of ART in any patient who had no evidence of newly acquired infection, evolution or | Drug-related Grade 3 & 4 adverse events using DAIDS table. Up until 50 weeks after last participant was | Not specifically defined, | HIV-1 RNA <400 copies/mL | NA |

| | | | | | | | |
|---|---|---|---|---|---|---|---|
| (CAMELIA ) | up to week 50 | | drug resistant TB, infection with a previously recognized pathogen, or side effects of ART. The adjudication of IRIS was not blinded" | enrolled (median 25 months). Numbers of events (not number of participants with ≥1 event). Death not specifically an SAE, although some of drug-related SAEs did lead to death. | extracted from KM graph up to week 50. [(4)] | at 50 weeks. | |
| Manosuthi et al. 2012 (TIME) | All case mortality at 1 year | "Major opportunistic infections and / or AIDS related malignancy" | "Patients were classified as having definite TB IRIS if they met criteria from a case definitions previously described by the International Network for the Study of HIV-associated IRIS" (Meintjes et al 2008). [27] | "Adverse events related to ART and anti-TB drugs were graded using division of AIDS table for grading of severity of adult and paediatric adverse events. Up to 96 weeks. Number of events (not number of participant with ≥1 event)." | Not defined, appears no participants LTFU. | NA | NA |
| Sinha et al 2012 | All cause morality at 12 months. | "WHO Stage 4 event" | Not specifically defined [(5)] | Results section reports incidence of IRIS and "other adverse events". Not specifically defined. | Not specifically defined. | HIV-1 RNA <10,000 copies / mL at any time after 6 months on ART. | Not specifically defined. |
| Mfinanga et al TB-HAART | All-cause mortality at 12 months. | "Rates of other opportunistic infections (as defined according to WHO staging system) and any event which leads to progression in WHO-defined clinical staging (e.g. extrapulmonary TB) over the course of 24 months." | We used criteria from the International Network for the Study of HIV-associated Immune Reconstitution Inflammatory Syndrome for classification of tuberculosis immune reconstitution inflammatory syndrome. (Meintjes et al).<br>As investigators, pharmacists and nurses were blinded until 6 months, presumably adjudication IRIS made without reference to ART status. | "Grade 3 and grade 4 drug related adverse events" up until 12 months. Includes IRIS as an adverse event. Number of events and numbers of people with >=1 event. No denominator of person-time provided. | Not specifically defined [(6)] | NA | NA [(7)] |
| Amogne et al (2015) | All cause mortality by 48 weeks. | "Category C disease listed by the CDC and WHO stage IV events" | "For the diagnosis of TB-associated IRIS, a case definition for use in resource limited settings was applied where at least one major criteria or two minor clinical criteria were required (Meintjes et al 2008). | Hepatotoxicity was graded using DAIDS table.<br><br>**NB. Only refers to hepatoxicity.** | "Lost to follow-up is defined as can't be reached of outcome traced" | HIV-1 RNA <400 copies/mL at 28 weeks. | NA |

| | | | | | | | |
|---|---|---|---|---|---|---|---|
| Merle et al 2020 (RAFA) | All cause morality at 12 months | NA | “TB IRIS as per Meintjes et al.” | Number of events (not number of participants with ≥1 event). Death included as an SAE. Full details of SAEs not available at time of writing this report (8) | Not specifically defined (8) | HIV-1 RNA < 1000 copies /mL at 18 months (9) | NA |

**ART:** antiretroviral therapy**. IRIS:** Immune Reconstitution Inflammatory Syndrome. **TB:** tuberculosis. **NA:** not applicable. **DAIDS:** Division of AIDS Table for Grading of Severity of Adult and Paediatric Adverse Events.

(1) Three deaths occurred in study, at week 4, week 20 and week 35. No further deaths occurred in 104 weeks of follow-up (the study-specified secondary outcome).

(2) Definition of AIDS-defining events not given, however details of clinical diagnoses are provided - 1 case of cryptococcal meningitis, 1 of non-tuberculous mycobacteraemia, 1 of disseminated Kaposi’s sarcoma.

(3) Also provides data on VL < 50 copies / mL

(4) Not just loss to follow-up, includes transfer out and withdrawl of consent. Overall, 12 people withdrew consent, 4 transferred HIV care and 12 were lost to follow-up over the entire course of study. Can extract from KM curve that 18 of these were before 50 weeks but not the reason for no longer being in study.

(5) IRIS isn’t defined, although authors’ report “all cases of IRIS were moderate and none required any interruption of HAART or management with steroids”.

(6) The authors give a clear definition for ART default (“missing two consecutive monthly ART refills”) which is relatively common (135 participants). Loss to follow-up not clearly defined, but they report number of people missing from final analysis (15 participants).

(7) No reported switching, ART default is reported and is defined as two or more consecutive missed monthly pick up of ART.

(8) SAE definitions not specifically defined in manuscript, protocol not available to us as we are preparing this report but will be an appendix once paper published.

Note that only a relatively small proportion of the participants had viral load measured (121 with viral load measured, at 18 months). Data also available on viral supression at 6 months (137 participants)

**Supplementary table 2A:** Outcomes per study (all CD4 counts)

| Paper | Year pub | CD4 | TB type | Early ART | Late ART | N randomised | | N with mortality outcome | | Death | | IRIS | | AIDS-defining events | |
|---|---|---|---|---|---|---|---|---|---|---|---|---|---|---|---|
| | | | | | | Early | Late | Early | Late | Early | Late | Early | Late | Early | Late |
| Shao (THIRST) | 2009 | <1200 TLC | Smear pos, any site (except CNS) | 2 weeks | 8 weeks | 35 | 35 | 35 | 35 | 2 / 35 (6%) | 1 / 35 (3%) | 0 / 35 (0%) | 0 / 35 (0%) | 1 / 35 (3%) | 2 / 35 (6%) |
| Abdool Karim (SAPiT) | 2011 | < 500 | Smear pos PTB | <4 weeks | 26+ weeks | 214 | 215 | 188 | 181 | 15 / 188 (8%) | 15 / 181 (8%) | 43 / 214 (20%) | 18 / 215 (8%) | 18 / 214 (8%) | 19 / 181 (9%) |
| Havlir (STRIDE) | 2011 | <250 | Probable or confirmed, any site | 2 weeks | 8 - 12 weeks | 405 | 401 | 368 | 376 | 31 / 368 (8%) | 37 / 376 (10%) | 43 / 405 (11%) | 19 / 401 (5%) | 26 / 405 (6%) | 37 / 376 (9%) |
| Blanc (CAMELIA) | 2011 | ≤ 200 | Smear pos, any site | 2 weeks | 8 weeks | 332 | 329 | 324 | 319 | 46 / 324 (14%) | 63 / 319 (20%) | 110 / 332 (33%) | 45 / 329 (14%) | NA | NA |
| Manosuthi (TIME) | 2012 | <350 | Probable or confirmed, any site | 4 weeks | 12 weeks | 79 | 77 | 79 | 77 | 6 / 79 (8%) | 5 / 77 (6%) | 26 / 79 (33%) | 15 / 77 (19%) | 9 / 79 (11%) | 14 / 77 (18%) |
| Sinha | 2012 | Any | Probable or confirmed, any site | 2 - 4 weeks | 8 - 12 weeks | 92 | 89 | 88 | 62 | 9 / 88 (10%) | 7 / 62 (11%) | 9 / 92 (10%) | 6 / 89 (7%) | 0 / 92 (0%) | 1 / 62 (1%) |
| Mfinanga (TB-HAART) | 2014 | ≥ 220 | Smear and culture positive PTB | 2 weeks | 6 months | 767 | 771 | 758 | 765 | 19 / 758 (3%) | 21 / 765 (3%) | 81 / 767 (11%) | 93 / 771 (12%) | NA | NA |
| Amogne 1vs4and8 | 2015 | <200 | Probable or confirmed, any site (except CNS) | 1 week | 4 weeks or 8 weeks | 163 | 315 | 137 | 270 | 27 / 137 (20%) | 37 / 270 (14%) | 16 / 163 (10%) | 6 / 315 (2%) | 12 / 163 (7%) | 27 / 270 (9%) |
| Amogne 1and4vs8 | 2015 | <200 | Probable or confirmed, any site (except CNS) | 1 week or 4 weeks | 8 weeks | 323 | 155 | 273 | 134 | 47 / 273 (17%) | 17 / 134 (13%) | 22 / 323 (7%) | 0 / 155 (0%) | 26 / 323 (8%) | 13 / 134 (8%) |
| Merle (RAFA) | Unpublish ed | > 50 | Micro confirmed pulmonary TB | 2 weeks | 8 weeks | 251 | 247 | 236 | 238 | 26 / 236 (11%) | 35 / 238 (15%) | 10 / 251 (4%) | 5 / 247 (2%) | NA | NA |

| Paper | Serious Adverse Events (SAEs) (all) | | Treatment-related SAEs / person-months | | Loss to follow-up | | Achieved HIV viral suppression | | ART switched / discontinued | |
|---|---|---|---|---|---|---|---|---|---|---|
| | Early | Late | Early | Late | Early | Late | Early | Late | Early | Late |
| Shao (THIRST) | 12 / 35 (34%) | 7 / 35 (20%) | NA | NA | 0 / 35 (0%) | 0 / 35 (0%) | 24 / 33 (73%) | 19 / 34 (54%) | 5 / 35 (14%) | 2 / 35 (6%) |
| Abdool Karim (SAPiT) | 112 / 214 (52%) | 107 / 215 (50%) | NA | NA | 46 / 214 (21%) | 57 / 215 (27%) | 147 / 159 (92%) | 130 / 147 (88%) | 16 / 214 (7%) | 10 / 215 (5%) |
| Havlir (STRIDE) | 177 / 405 (44%) | 190 / 401 (47%) | NA | NA | 37 / 405 (9%) | 25 / 401 (6%) | 293 / 331 (89%) | 301 / 332 (91%) | 14 / 405 (3%) | 7 / 401 (2%) |
| Blanc (CAMELIA) | 251 / 332 (76%) | 245 / 329 (74%) | 251 / 8567 (76%) | 245 / 7632 (74%) | 6 / 332 (2%) | 6 / 329 (2%) | 263 / 273 (96%) | 238 / 247 (96%) | NA | NA |
| Manosuthi (TIME) | 19 / 79 (24%) | 19 / 77 (25%) | 19 / 79 (24%) | 19 / 77 (25%) | NA | NA | NA | NA | NA | NA |
| Sinha | 21 / 92 (23%) | 14 / 89 (16%) | NA | NA | 15 / 92 (16%) | 28 / 89 (31%) | NA | NA | 4 / 92 (4%) | 3 / 89 (3%) |
| Mfinanga (TB-HAART) | 149 / 767 (19%) | 174 / 771 (23%) | NA | NA | 9 / 767 (1%) | 6 / 771 (1%) | NA | NA | NA | NA |
| Amogne 1vs4and8 | 27 / 163 (17%) | 44 / 315 (14%) | 27 / 1296 (17%) | 44 / 2780 (14%) | 26 / 163 (16%) | 45 / 315 (14%) | 50 / 53 (94%) | 150 / 167 (90%) | NA | NA |

| Paper | Serious Adverse Events (SAEs) (all) | | Treatment-related SAEs / person-months | | Loss to follow-up | | Achieved HIV viral suppression | | ART switched / discontinued | |
|---|---|---|---|---|---|---|---|---|---|---|
| | Early | Late | Early | Late | Early | Late | Early | Late | Early | Late |
| Amogne 1and4vs8 | 50 / 323 (15%) | 21 / 155 (14%) | 50 / 2676 (15%) | 21 / 1400 (14%) | 50 / 323 (15%) | 21 / 155 (14%) | 117 / 127 (92%) | 83 / 93 (89%) | NA | NA |
| Merle (RAFA) | 22 / 251 (9%) | 14 / 247 (6%) | NA | NA | 15 / 251 (6%) | 9 / 247 (4%) | 54 / 72 (75%) | 38 / 49 (78%) | NA | NA |

**Supplementary table 2B:** Outcomes per study (CD <=50)

| Paper | Year pub | CD4 subgroup | TB type | Early ART | Late ART | N randomised | | N with mortality outcome | | Death | | IRIS | | AIDS-defining events | |
|---|---|---|---|---|---|---|---|---|---|---|---|---|---|---|---|
| | | | | | | Early | Late | Early | Late | Early | Late | Early | Late | Early | Late |
| Abdool Karim (SAPiT) | 2011 | < 50 | Smear pos PTB | <4 weeks | 26+ weeks | 37 | 35 | 37 | 35 | 3 / 37 (8%) | 7 / 35 (20%) | 14 / 37 (38%) | 4 / 35 (11%) | 4 / 37 (11%) | 10 / 35 (29%) |
| Havlir (STRIDE) | 2011 | < 50 | Probable or confirmed, any site | 2 weeks | 8 - 12 weeks | 144 | 141 | NA | NA | 14 / 144 (10%) | 24 / 141 (17%) | 26 / 144 (18%) | 7 / 141 (5%) | 11 / 144 (8%) | 23 / NA (16%) |
| Blanc (CAMELIA) | 2011 | <= 50 | Smear pos, any site | 2 weeks | 8 weeks | 237 | 238 | NA | NA | 39 / 237 (16%) | 51 / 238 (21%) | 82 / 237 (35%) | 24 / 238 (10%) | NA | NA |
| Manosuthi (TIME) | 2012 | < 50 | Probable or confirmed, any site | 4 weeks | 12 weeks | 46 | 38 | 46 | 38 | 4 / 46 (9%) | 5 / 38 (13%) | 15 / 46 (33%) | 8 / 38 (21%) | NA | NA |
| Amogne 1vs4and8 | 2015 | <= 50 | Probable or confirmed, any site (except CNS) | 1 week | 4 weeks or 8 weeks | 59 | 89 | 59 | 89 | 16 / 59 (27%) | 21 / 89 (24%) | 16 / 59 (27%) | 6 / 89 (7%) | 12 / 59 (20%) | 27 / 89 (30%) |
| Amogne 1and4vs8 | 2015 | <= 50 | Probable or confirmed, any site (except CNS) | 1 week or 4 weeks | 8 weeks | 108 | 40 | 108 | 40 | 27 / 108 (25%) | 10 / 40 (25%) | 22 / 108 (20%) | 0 / 40 (0%) | 26 / 108 (24%) | 13 / 40 (32%) |

| Paper | Serious Adverse Events (SAEs) (all) | | Treatment-related SAEs / person-months | | Loss to follow-up | | Achieved HIV viral suppression | | ART switched / discontinued | |
|---|---|---|---|---|---|---|---|---|---|---|
| | Early | Late | Early | Late | Early | Late | Early | Late | Early | Late |
| Abdool Karim (SAPiT) | NA | NA | NA | NA | NA | NA | 30 / 32 (94%) | 23 / 27 (85%) | NA | NA |
| Havlir (STRIDE) | NA | NA | NA | NA | NA | NA | NA | NA | NA | NA |
| Blanc (CAMELIA) | NA | NA | NA | NA | NA | NA | NA | NA | NA | NA |
| Manosuthi (TIME) | 15 / 46 (33%) | 11 / 38 (29%) | 15 / 552 (33%) | 11 / 456 (29%) | 0 / 46 (0%) | 0 / 38 (0%) | NA | NA | NA | NA |
| Amogne 1vs4and8 | 20 / 59 (34%) | 12 / 89 (13%) | NA | NA | 10 / 59 (17%) | 12 / 89 (13%) | 16 / 16 (100%) | 40 / 41 (98%) | NA | NA |
| Amogne 1and4vs8 | 25 / 108 (23%) | 7 / 40 (18%) | NA | NA | 17 / 108 (16%) | 5 / 40 (12%) | 36 / 37 (97%) | 20 / 20 (100%) | NA | NA |

**Supplementary table 2C:** Outcomes per study (CD4 > 50)

| Paper | Year pub | CD4 | TB type | Early ART | Late ART | N randomised | | N with mortality outcome | | Death | | IRIS | | AIDS-defining events | |
|---|---|---|---|---|---|---|---|---|---|---|---|---|---|---|---|
| | | | | | | Early | Late | Early | Late | Early | Late | Early | Late | Early | Late |
| Abdool Karim (SAPiT) | 2011 | >=50 | Smear pos PTB | <4 weeks | 26+ weeks | 177 | 180 | 177 | 180 | 12 / 177 (7%) | 8 / 180 (4%) | 29 / 177 (16%) | 14 / 180 (8%) | 14 / 177 (8%) | 9 / 180 (5%) |
| Havlir (STRIDE) | 2011 | >=50 | Probable or confirmed, any site | 2 weeks | 8 - 12 weeks | 261 | 260 | NA | NA | 17 / 261 (7%) | 13 / 260 (5%) | 17 / 261 (7%) | 12 / 260 (5%) | 15 / 261 (6%) | 14 / 260 (5%) |
| Blanc (CAMELIA) | 2011 | >50 | Smear pos, any site | 2 weeks | 8 weeks | 95 | 91 | NA | NA | 7 / 95 (7%) | 12 / 91 (13%) | 28 / 95 (29%) | 11 / 91 (12%) | NA | NA |
| Manosuthi (TIME) | 2012 | >=50 | Probable or confirmed, any site | 4 weeks | 12 weeks | 33 | 39 | 33 | 39 | 2 / 33 (6%) | 0 / 39 (0%) | 11 / 33 (33%) | 7 / 39 (18%) | NA | NA |
| Mfinanga (TB-HAART) | 2014 | All >=220 | Smear and culture positive PTB | 2 weeks | 6 months | 767 | 771 | 684 | 719 | 19 / 767 (2%) | 21 / 771 (3%) | 81 / 767 (11%) | 93 / 771 (12%) | NA | NA |
| Amogne 1vs4and8 | 2015 | >50 | Probable or confirmed, any site (except CNS) | 1 week | 8 weeks | 104 | 226 | 104 | 226 | 11 / 104 (11%) | 16 / 226 (7%) | 0 / 104 (0%) | 0 / 226 (0%) | 0 / 104 (0%) | 0 / 226 (0%) |
| Amogne 1and4vs8 | 2015 | >50 | Probable or confirmed, any site (except CNS) | 1 week or 4 weeks | 8 weeks | 215 | 115 | 215 | 115 | 20 / 215 (9%) | 7 / 115 (6%) | 0 / 215 (0%) | 0 / 115 (0%) | 0 / 215 (0%) | 0 / 115 (0%) |
| Merle (RAFA) | Unpublished | All >= 50 | Micro confirmed pulmonary TB | 2 weeks | 8 weeks | 251 | 247 | 236 | 238 | 26 / 251 (10%) | 35 / 247 (14%) | 10 / 251 (4%) | 5 / 247 (2%) | NA | NA |

| Paper | Serious Adverse Events (SAEs) (all) | | Treatment-related SAEs / person-months | | Loss to follow-up | | Achieved HIV viral suppression | | ART switched / discontinued | |
|---|---|---|---|---|---|---|---|---|---|---|
| | Early | Late | Early | Late | Early | Late | Early | Late | Early | Late |
| Abdool Karim (SAPiT) | Early | Late | Early | Late | Early | Late | Early | Late | Early | Late |
| Havlir (STRIDE) | NA | NA | NA | NA | NA | NA | NA | NA | NA | NA |
| Blanc (CAMELIA) | NA | NA | NA | NA | NA | NA | NA | NA | NA | NA |
| Manosuthi (TIME) | 4 / 33 (12%) | 8 / 39 (21%) | 4 / 264 (12%) | 8 / 468 (21%) | 0 / 33 (0%) | 0 / 39 (0%) | NA | NA | NA | NA |
| Mfinanga (TB-HAART) | 149 / 767 (19%) | 174 / 771 (23%) | NA | NA | 83 / 767 (11%) | 52 / 771 (7%) | NA | NA | NA | NA |
| Amogne 1vs4and8 | 7 / 104 (7%) | 32 / 226 (14%) | NA | NA | 17 / 104 (16%) | 32 / 226 (14%) | 34 / 37 (92%) | 110 / 126 (87%) | NA | NA |
| Amogne 1and4vs8 | 25 / 215 (12%) | 14 / 115 (12%) | NA | NA | 35 / 215 (16%) | 14 / 115 (12%) | 81 / 90 (90%) | 63 / 73 (86%) | NA | NA |
| Merle (RAFA) | 22 / 251 (9%) | 14 / 247 (6%) | NA | NA | 15 / 251 (6%) | 9 / 247 (4%) | 54 / 72 (75%) | 38 / 49 (78%) | NA | NA |
