# Supplementary material for: What is the optimum time to start antiretroviral therapy in people with HIV and tuberculosis coinfection? A systematic review and meta‐analysis
Source: J Int AIDS Soc. 2021 Jul 21;24(7):e25772. doi: 10.1002/jia2.25772 (PMC8294654; doi:10.1002/jia2.25772)

Appendix 3: Forest plots, timing of ART in TB/HIV

Comparison A is ART <=2 weeks after TB treatment compared to ART >2 weeks and <= 8 weeks after TB treatment. Four studies using ART timepoints that permitted this comparison. Comparison B is ART <=4 weeks after TB treamtent compared to ART > 4 weeks after TB treatment.

Three studies (Amogne, SAPiT and TIME) had data disaggregated by CD4 count, two studies (RAFA and TB-HAART) only included people with CD4 counts > 50 cells/mm3, two further studies (CAMELIA and STRIDE) provided some CD4-disaggregated data directly from authors.

Note that Amogne et al is a study that used three timepoints (1 week, 4 weeks and 8 weeks). For comparison A the 4 weeks and 8 week groups are combined ("Amogne 1vs4and8"), for comparison B the 1 and 4 week groups are combined ("Amogne 1and4vs8").

The SAPiT trial initially randomised people into three arms (4 weeks, 8-12 weeks and 26 weeks). The 26 week group was stopped early due to signal of harm. SAPiT data are reported in two primary manuscripts, one comparing combined 4 and 8-12 weeks group to 26 weeks group and one comparing 4 and 8-12 weeks groups to each other. All SAPiT data in these meta-analyses pertain to the comparison of 4 weeks vs. 8-12 weeks (2011 NEJM paper).

Not all studies disaggregated data by CD4 criteria and some studies allowed disaggregation of some outcomes but not others, so the graphs of low CD4 (<= 50 cells) and higher CD4 (> 50 cells) do not represent all the available data (THIRST and Sinha et al do not have any CD4 disaggregated data and so are not present in analyses by CD4 strata),

All summary estimates summary estimates are from random effects meta-regression models using package 'meta' in R statistical software.

Death

All CD4 counts

Comparison A (ART <=2 weeks vs. ART >2 weeks and <=8 weeks)

	ART =<2 wks		ART 2−8 wks			
**Study**	**Events**	**Total**	**Events**	**Total**	**Weight**	**RD [95% CI]**
Shao (THIRST)	2	35	1	35	17.3%	0.03 [−0.07; 0.12]
Blanc (CAMELIA)	46	332	63	329	29.0%	−0.05 [−0.11; 0.00]
Amogne 1vs4and8	27	163	37	315	25.1%	0.05 [−0.02; 0.12]
Merle (RAFA)	26	251	35	247	28.6%	−0.04 [−0.10; 0.02]
**Total (95% CI)**	**101**	**781**	**136**	**926**	**100.0%**	**−0.01 [−0.06; 0.04]**

Heterogeneity: $Tau^2 = 0.0014$; $Chi^2 = 6.61$, df = 3 (P = 0.09); $I^2 = 55\%$

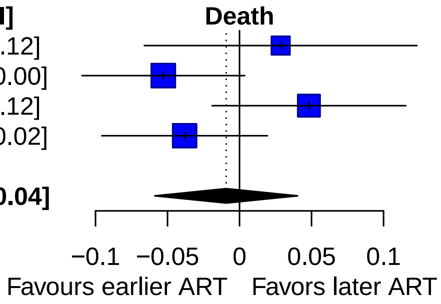

Comparison B (ART <=4 weeks vs. ART > 4 weeks)

	ART =<4 wks		ART > 4 wks			
**Study**	**Events**	**Total**	**Events**	**Total**	**Weight**	**RD [95% CI]**
Shao (THIRST)	2	35	1	35	1.8%	0.03 [−0.07; 0.12]
Blanc (CAMELIA)	46	332	63	329	4.9%	−0.05 [−0.11; 0.00]
Abdool Karim (SAPiT)	15	214	15	215	6.8%	0.00 [−0.05; 0.05]
Havlir (STRIDE)	31	405	37	401	10.7%	−0.02 [−0.05; 0.02]
Sinha	9	92	7	89	2.3%	0.02 [−0.06; 0.10]
Manosuthi (TIME)	6	79	5	77	2.4%	0.01 [−0.07; 0.09]
Mfinanga (TB−HAART)	19	767	21	771	62.3%	−0.00 [−0.02; 0.01]
Amogne 1and4vs8	47	323	17	155	4.0%	0.04 [−0.03; 0.10]
Merle (RAFA)	26	251	35	247	4.8%	−0.04 [−0.10; 0.02]
**Total (95% CI)**	**201**	**2498**	**201**	**2319**	**100.0%**	**−0.00 [−0.02; 0.01]**

Heterogeneity: $Tau^2 = 0$; $Chi^2 = 7.74$, df = 8 (P = 0.46); $I^2 = 0\%$

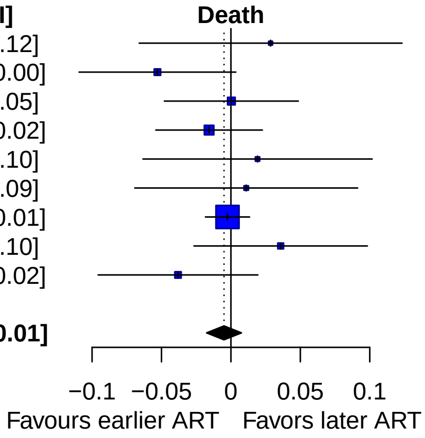

Low CD4 counts (CD4 =<50)

Comparison A (ART <=2 weeks vs. ART >2 weeks and <=8 weeks)

	ART =<2 wks		ART 2−8 wks			
**Study**	**Events**	**Total**	**Events**	**Total**	**Weight**	**RD [95% CI]**
Amogne 1vs4and8	16	59	21	89	21.9%	0.04 [−0.11; 0.18]
Blanc (CAMELIA)	39	237	51	238	78.1%	−0.05 [−0.12; 0.02]
**Total (95% CI)**	**55**	**296**	**72**	**327**	**100.0%**	**−0.03 [−0.10; 0.04]**

Heterogeneity: $Tau^2 = 0.0003$; $Chi^2 = 1.09$, df = 1 (P = 0.30); $I^2 = 8\%$

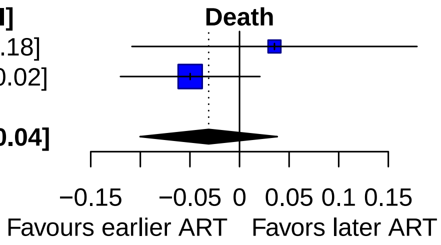

Comparison B (ART <=4 weeks vs. ART > 4 weeks)

	ART =<4 wks		ART > 4 wks			
**Study**	**Events**	**Total**	**Events**	**Total**	**Weight**	**RD [95% CI]**
Abdool Karim (SAPiT)	3	37	7	35	7.9%	−0.12 [−0.28; 0.04]
Havlir (STRIDE)	14	144	24	141	32.4%	−0.07 [−0.15; 0.01]
Amogne 1and4vs8	27	108	10	40	8.1%	0.00 [−0.16; 0.16]
Blanc (CAMELIA)	39	237	51	238	40.5%	−0.05 [−0.12; 0.02]
Manosuthi (TIME)	4	46	5	38	11.0%	−0.04 [−0.18; 0.09]
**Total (95% CI)**	**87**	**572**	**97**	**492**	**100.0%**	**−0.06 [−0.10; −0.01]**

Heterogeneity: $Tau^2 = 0$; $Chi^2 = 1.34$, df = 4 (P = 0.86); $I^2 = 0\%$

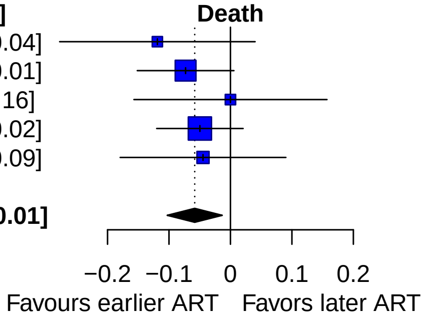

High CD4 counts (CD4 > 50)

Comparison A (ART <=2 weeks vs. ART >2 weeks and <=8 weeks)

	ART =<2 wks		ART 2−8 wks			
**Study**	**Events**	**Total**	**Events**	**Total**	**Weight**	**RD [95% CI]**
Amogne 1vs4and8	11	104	16	226	34.2%	0.03 [−0.03; 0.10]
Blanc (CAMELIA)	7	95	12	91	25.4%	−0.06 [−0.15; 0.03]
Merle (RAFA)	26	251	35	247	40.3%	−0.04 [−0.10; 0.02]
**Total (95% CI)**	**44**	**450**	**63**	**564**	**100.0%**	**−0.02 [−0.07; 0.04]**

Heterogeneity: $Tau^2 = 0.0010$; $Chi^2 = 3.64$, df = 2 (P = 0.16); $I^2 = 45\%$

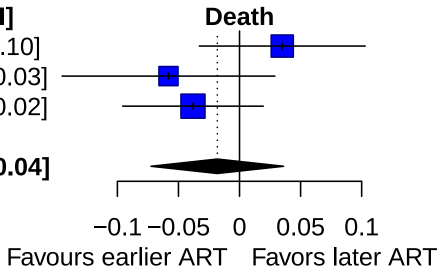

Comparison B (ART <=4 weeks vs. ART > 4 weeks)

	ART =<4 wks		ART > 4 wks			
**Study**	**Events**	**Total**	**Events**	**Total**	**Weight**	**RD [95% CI]**
Abdool Karim (SAPiT)	12	177	8	180	12.8%	0.02 [−0.02; 0.07]
Havlir (STRIDE)	17	261	13	260	16.7%	0.02 [−0.02; 0.06]
Mfinanga (TB−HAART)	19	767	21	771	43.6%	−0.00 [−0.02; 0.01]
Amogne 1and4vs8	20	215	7	115	9.1%	0.03 [−0.03; 0.09]
Blanc (CAMELIA)	7	95	12	91	4.5%	−0.06 [−0.15; 0.03]
Merle (RAFA)	26	251	35	247	9.4%	−0.04 [−0.10; 0.02]
Manosuthi (TIME)	2	33	0	39	3.9%	0.06 [−0.03; 0.15]
**Total (95% CI)**	**103**	**1799**	**96**	**1703**	**100.0%**	**0.00 [−0.02; 0.02]**

Heterogeneity: $Tau^2 = 0.0002$; $Chi^2 = 7.79$, df = 6 (P = 0.25); $I^2 = 23\%$

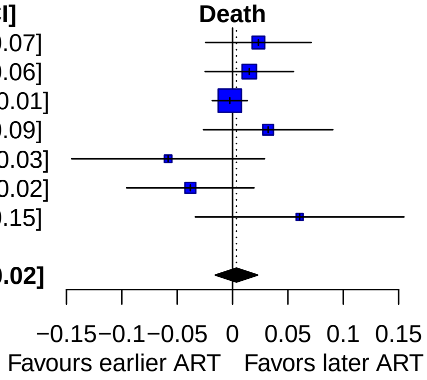

IRIS

All CD4 counts

Comparison A (ART <=2 weeks vs. ART >2 weeks and <=8 weeks)

	ART =<2 wks		ART 2−8 wks			
**Study**	**Events**	**Total**	**Events**	**Total**	**Weight**	**RD [95% CI]**
Shao (THIRST)	0	35	0	35	24.7%	0.00 [−0.05; 0.05]
Blanc (CAMELIA)	110	332	45	329	24.1%	0.19 [0.13; 0.26]
Amogne 1vs4and8	16	163	6	315	25.1%	0.08 [0.03; 0.13]
Merle (RAFA)	10	251	5	247	26.1%	0.02 [−0.01; 0.05]
**Total (95% CI)**	**136**	**781**	**56**	**926**	**100.0%**	**0.07 [−0.03; 0.17]**

Heterogeneity: $Tau^2 = 0.0095$; $Chi^2 = 53.76$, df = 3 ($P < 0.01$); $I^2 = 94\%$

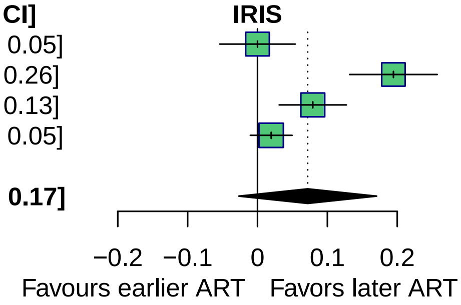

Comparison B (ART <=4 weeks vs. ART > 4 weeks)

	ART =<4 wks		ART > 4 wks			
**Study**	**Events**	**Total**	**Events**	**Total**	**Weight**	**RD [95% CI]**
Shao (THIRST)	0	35	0	35	11.4%	0.00 [−0.05; 0.05]
Blanc (CAMELIA)	110	332	45	329	10.6%	0.19 [0.13; 0.26]
Abdool Karim (SAPiT)	43	214	18	215	10.4%	0.12 [0.05; 0.18]
Havlir (STRIDE)	43	405	19	401	12.9%	0.06 [0.02; 0.10]
Sinha	9	92	6	89	9.1%	0.03 [−0.05; 0.11]
Manosuthi (TIME)	26	79	15	77	5.4%	0.13 [0.00; 0.27]
Mfinanga (TB−HAART)	81	767	93	771	13.3%	−0.02 [−0.05; 0.02]
Amogne 1and4vs8	22	323	0	155	13.5%	0.07 [0.04; 0.10]
Merle (RAFA)	10	251	5	247	13.4%	0.02 [−0.01; 0.05]
**Total (95% CI)**	**344**	**2498**	**201**	**2319**	**100.0%**	**0.06 [0.02; 0.10]**

Heterogeneity: $Tau^2 = 0.0028$; $Chi^2 = 53.76$, df = 8 ($P < 0.01$); $I^2 = 85\%$

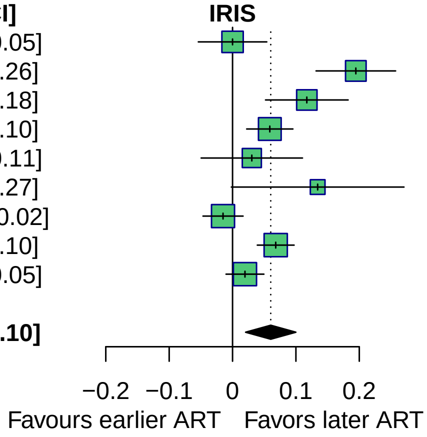

Low CD4 counts (CD4 =<50)

Comparison A (ART <=2 weeks vs. ART >2 weeks and <=8 weeks)

	ART =<2 wks		ART 2−8 wks			
**Study**	**Events**	**Total**	**Events**	**Total**	**Weight**	**RD [95% CI]**
Amogne 1vs4and8	16	59	6	89	24.8%	0.20 [0.08; 0.33]
Blanc (CAMELIA)	82	237	24	238	75.2%	0.25 [0.17; 0.32]
**Total (95% CI)**	**98**	**296**	**30**	**327**	**100.0%**	**0.23 [0.17; 0.30]**

Heterogeneity: $Tau^2 = 0$; $Chi^2 = 0.32$, df = 1 (P = 0.57); $I^2 = 0\%$

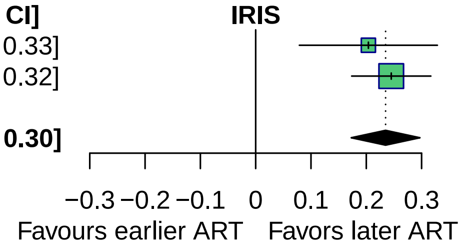

Comparison B (ART <=4 weeks vs. ART > 4 weeks)

	ART =<4 wks		ART > 4 wks			
**Study**	**Events**	**Total**	**Events**	**Total**	**Weight**	**RD [95% CI]**
Abdool Karim (SAPiT)	14	37	4	35	7.6%	0.26 [0.08; 0.45]
Havlir (STRIDE)	26	144	7	141	29.5%	0.13 [0.06; 0.20]
Amogne 1and4vs8	22	108	0	40	25.5%	0.20 [0.12; 0.29]
Blanc (CAMELIA)	82	237	24	238	29.8%	0.25 [0.17; 0.32]
Manosuthi (TIME)	15	46	8	38	7.7%	0.12 [−0.07; 0.30]
**Total (95% CI)**	**159**	**572**	**43**	**492**	**100.0%**	**0.19 [0.14; 0.25]**

Heterogeneity: $Tau^2 = 0.0014$; $Chi^2 = 6.25$, df = 4 (P = 0.18); $I^2 = 36\%$

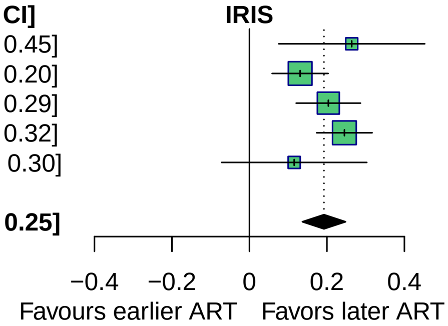

NB. For Blanc 2011 (CAMELIA) trial, CD4 count groupings of incident IRIS are based on baseline CD4 count, not screening CD4 count. For Amogne et al (2015) counts by CD4 categories calculated from the supplementary data file provided with manuscript.

High CD4 counts (CD4 > 50)

Comparison A (ART <=2 weeks vs. ART >2 weeks and <=8 weeks)

	ART =<2 wks		ART 2−8 wks			
**Study**	**Events**	**Total**	**Events**	**Total**	**Weight**	**RD [95% CI]**
Amogne 1vs4and8	0	104	0	226	38.6%	0.00 [−0.01; 0.01]
Blanc (CAMELIA)	28	95	11	91	24.0%	0.17 [0.06; 0.29]
Merle (RAFA)	10	251	5	247	37.4%	0.02 [−0.01; 0.05]
**Total (95% CI)**	**38**	**450**	**16**	**564**	**100.0%**	**0.05 [−0.04; 0.14]**

Heterogeneity: $Tau^2 = 0.0054$; $Chi^2 = 41.93$, df = 2 ($P < 0.01$); $I^2 = 95\%$

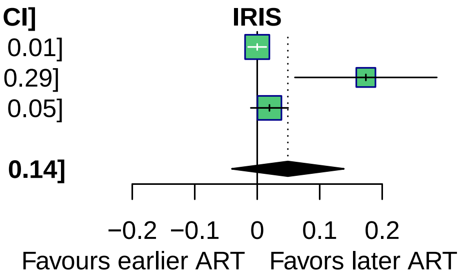

Comparison B (ART <=4 weeks vs. ART > 4 weeks)

	ART =<4 wks		ART > 4 wks			
**Study**	**Events**	**Total**	**Events**	**Total**	**Weight**	**RD [95% CI]**
Abdool Karim (SAPiT)	29	177	14	180	11.6%	0.09 [0.02; 0.15]
Havlir (STRIDE)	17	261	12	260	17.6%	0.02 [−0.02; 0.06]
Mfinanga (TB−HAART)	81	767	93	771	19.5%	−0.02 [−0.05; 0.02]
Amogne 1and4vs8	0	215	0	115	23.1%	0.00 [−0.01; 0.01]
Blanc (CAMELIA)	28	95	11	91	5.9%	0.17 [0.06; 0.29]
Merle (RAFA)	10	251	5	247	19.9%	0.02 [−0.01; 0.05]
Manosuthi (TIME)	11	33	7	39	2.3%	0.15 [−0.05; 0.35]
**Total (95% CI)**	**176**	**1799**	**142**	**1703**	**100.0%**	**0.03 [0.00; 0.06]**

Heterogeneity: $Tau^2 = 0.0011$; $Chi^2 = 25.88$, df = 6 ($P < 0.01$); $I^2 = 77\%$

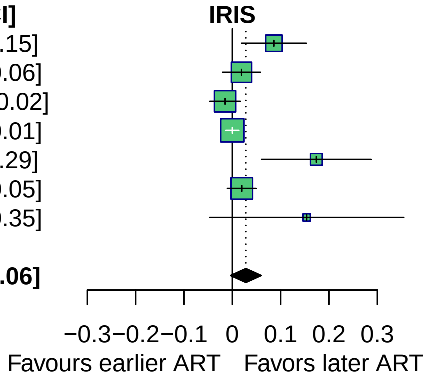

Aids Defining Events

All CD4 counts

Comparison A (ART <=2 weeks vs. ART >2 weeks and <=8 weeks)

	ART =<2 wks		ART 2−8 wks			
**Study**	**Events**	**Total**	**Events**	**Total**	**Weight**	**RD [95% CI]**
Shao (THIRST)	1	35	2	35	22.2%	−0.03 [−0.12; 0.07]
Amogne 1vs4and8	12	163	27	315	77.8%	−0.01 [−0.06; 0.04]
**Total (95% CI)**	**13**	**198**	**29**	**350**	**100.0%**	**−0.02 [−0.06; 0.03]**

Heterogeneity: $Tau^2 = 0$; $Chi^2 = 0.09$, df = 1 (P = 0.76); $I^2 = 0\%$

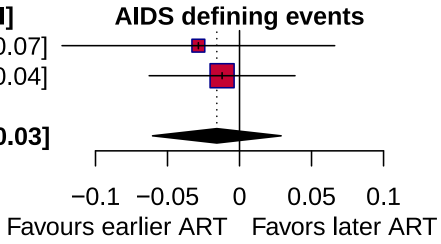

Comparison B (ART <=4 weeks vs. ART > 4 weeks)

	ART =<4 wks		ART > 4 wks			
**Study**	**Events**	**Total**	**Events**	**Total**	**Weight**	**RD [95% CI]**
Shao (THIRST)	1	35	2	35	4.1%	−0.03 [−0.12; 0.07]
Abdool Karim (SAPiT)	18	214	19	215	13.0%	−0.00 [−0.06; 0.05]
Havlir (STRIDE)	26	405	37	401	26.8%	−0.03 [−0.07; 0.01]
Sinha	0	92	1	89	39.9%	−0.01 [−0.04; 0.02]
Manosuthi (TIME)	9	79	14	77	3.0%	−0.07 [−0.18; 0.04]
Amogne 1and4vs8	26	323	13	155	13.2%	−0.00 [−0.06; 0.05]
**Total (95% CI)**	**80**	**1148**	**86**	**972**	**100.0%**	**−0.02 [−0.04; 0.00]**

Heterogeneity: $Tau^2 = 0$; $Chi^2 = 1.94$, df = 5 (P = 0.86); $I^2 = 0\%$

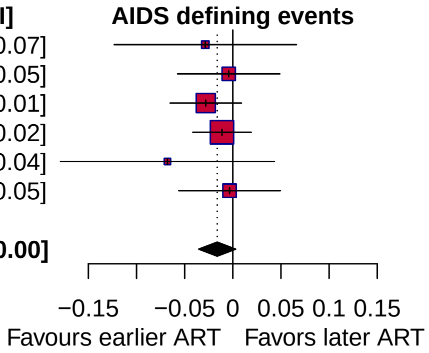

Low CD4 counts (CD4 =<50)

Comparison A (ART <=2 weeks vs. ART >2 weeks and <=8 weeks)

	ART =<2 wks		ART 2−8 wks		
**Study**	**Events**	**Total**	**Events**	**Total**	**RD [95% CI]**
Amogne 1vs4and8	12	59	27	89	−0.10 [−0.24; 0.04]

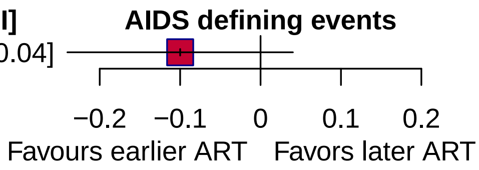

Comparison B (ART <=4 weeks vs. ART > 4 weeks)

	ART =<4 wks		ART > 4 wks			
**Study**	**Events**	**Total**	**Events**	**Total**	**Weight**	**RD [95% CI]**
Abdool Karim (SAPiT)	4	37	10	35	12.6%	−0.18 [−0.36; 0.00]
Havlir (STRIDE)	11	144	23	141	72.7%	−0.09 [−0.16; −0.01]
Amogne 1and4vs8	26	108	13	40	14.8%	−0.08 [−0.25; 0.08]
**Total (95% CI)**	**41**	**289**	**46**	**216**	**100.0%**	**−0.10 [−0.16; −0.03]**

Heterogeneity: $Tau^2 = 0$; $Chi^2 = 0.87$, df = 2 (P = 0.65); $I^2 = 0\%$

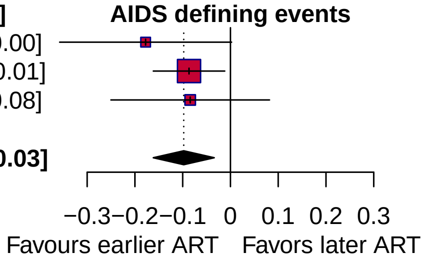

High CD4 counts (CD4 > 50)

Comparison A (ART <=2 weeks vs. ART >2 weeks and <=8 weeks)

	ART =<2 wks		ART 2−8 wks			
**Study**	**Events**	**Total**	**Events**	**Total**	**RD [95% CI]**	**AIDS defining events**
Amogne 1vs4and8	0	104	0	226	0.00 [−0.01; 0.01]	

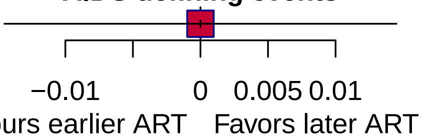

Comparison B (ART <=4 weeks vs. ART > 4 weeks)

	ART =<4 wks		ART > 4 wks				
**Study**	**Events**	**Total**	**Events**	**Total**	**Weight**	**RD [95% CI]**	**AIDS defining events**
Abdool Karim (SAPiT)	14	177	9	180	14.2%	0.03 [−0.02; 0.08]	
Havlir (STRIDE)	15	261	14	260	21.5%	0.00 [−0.04; 0.04]	
Amogne 1and4vs8	0	215	0	115	64.3%	0.00 [−0.01; 0.01]	
**Total (95% CI)**	**29**	**653**	**23**	**555**	**100.0%**	**0.00 [−0.02; 0.03]**	

Heterogeneity: $Tau^2 = 0.0001$; $Chi^2 = 2.91$, df = 2 (P = 0.23); $I^2 = 31\%$

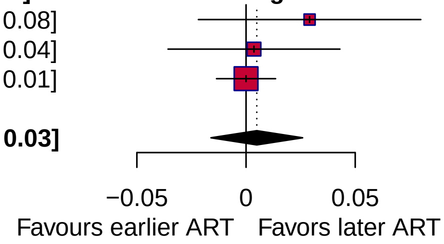

Serious Adverse Events

Note that Serious Adverse Events (SAEs) have very different definitions in each study and comparisons may not be meaningful. Some studies include IRIS and AIDS defining events as SAEs, whilst others don't. Most studies report total numbers of SAEs, not whether an individual person had an SAE or not (ie. a single person can have more than one SAE). One study (Amogne) only reports hepatoxicity and not other SAEs.

This meta-analysis is only for the two studies that specifically report **treatment assosciated** SAEs and considers incidence of treatment-related serious adverse events by per person-years of observation.

All CD4 counts

Comparison B (ART <=4 weeks vs. ART > 4 weeks)

	ART =<4 wks		ART > 4 wks			
**Study**	**Events**	**Time**	**Events**	**Time**	**Weight**	**IRD [95% CI]**
Blanc (CAMELIA)	251	714	245	636	85.1%	−0.03 [−0.10; 0.03]
Manosuthi (TIME)	19	79	19	77	14.9%	−0.01 [−0.16; 0.15]
**Total (95% CI)**	**270**		**264**		**100.0%**	**−0.03 [−0.09; 0.03]**

Heterogeneity: $Tau^2 = 0$; $Chi^2 = 0.10$, df = 1 (P = 0.75); $I^2 = 0\%$

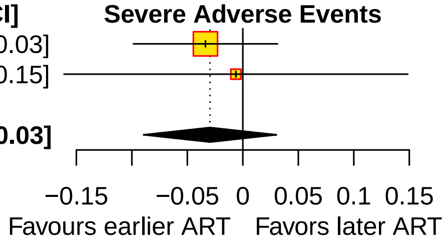

VL supression

All CD4 counts

Comparison A (ART <=2 weeks vs. ART >2 weeks and <=8 weeks)

	ART =<2 wks		ART 2−8 wks			
**Study**	**Events**	**Total**	**Events**	**Total**	**Weight**	**RD [95% CI]**
Shao (THIRST)	24	33	19	34	3.5%	0.17 [−0.06; 0.39]
Blanc (CAMELIA)	263	273	238	247	65.3%	−0.00 [−0.03; 0.03]
Amogne 1vs4and8	50	53	150	167	23.9%	0.05 [−0.03; 0.12]
Merle (RAFA)	54	72	38	49	7.3%	−0.03 [−0.18; 0.13]
**Total (95% CI)**	**391**	**431**	**445**	**497**	**100.0%**	**0.01 [−0.03; 0.06]**

Heterogeneity: $Tau^2 = 0.0005$; $Chi^2 = 3.70$, df = 3 (P = 0.30); $I^2 = 19\%$

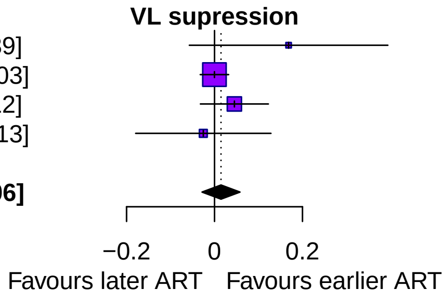

Comparison B (ART <=4 weeks vs. ART > 4 weeks)

	ART =<4 wks		ART > 4 wks			
**Study**	**Events**	**Total**	**Events**	**Total**	**Weight**	**RD [95% CI]**
Shao (THIRST)	24	33	19	34	1.1%	0.17 [−0.06; 0.39]
Blanc (CAMELIA)	263	273	238	247	51.0%	−0.00 [−0.03; 0.03]
Abdool Karim (SAPiT)	147	159	130	147	12.3%	0.04 [−0.03; 0.11]
Havlir (STRIDE)	293	331	301	332	24.7%	−0.02 [−0.07; 0.03]
Amogne 1and4vs8	117	127	83	93	8.7%	0.03 [−0.05; 0.11]
Merle (RAFA)	54	72	38	49	2.3%	−0.03 [−0.18; 0.13]
**Total (95% CI)**	**898**	**995**	**809**	**902**	**100.0%**	**0.00 [−0.02; 0.03]**

Heterogeneity: $Tau^2 < 0.0001$; $Chi^2 = 5.01$, df = 5 (P = 0.41); $I^2 = 0\%$

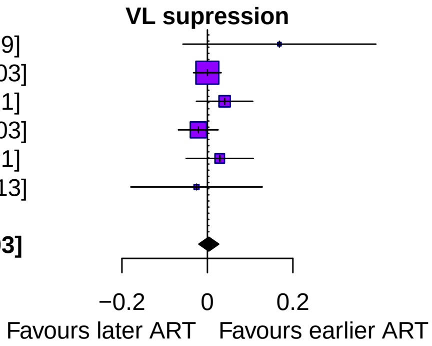

Denominator is all those who had VL measured, not all those randomised.

Low CD4 counts (CD4 =<50)

Comparison A (ART <=2 weeks vs. ART >2 weeks and <=8 weeks)

	ART =<2 wks		ART 2−8 wks		
**Study**	**Events**	**Total**	**Events**	**Total**	**RD [95% CI]**
Amogne 1vs4and8	16	16	40	41	0.02 [−0.07; 0.12]

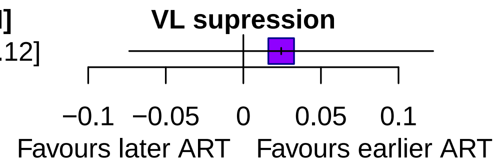

Comparison B (ART <=4 weeks vs. ART > 4 weeks)

	ART =<4 wks		ART > 4 wks			
**Study**	**Events**	**Total**	**Events**	**Total**	**Weight**	**RD [95% CI]**
Abdool Karim (SAPiT)	30	32	23	27	38.0%	0.09 [−0.07; 0.24]
Amogne 1and4vs8	36	37	20	20	62.0%	−0.03 [−0.12; 0.06]
**Total (95% CI)**	**66**	**69**	**43**	**47**	**100.0%**	**0.02 [−0.11; 0.14]**

Heterogeneity: $Tau^2 = 0.0048$; $Chi^2 = 2.13$, df = 1 (P = 0.14); $I^2 = 53\%$

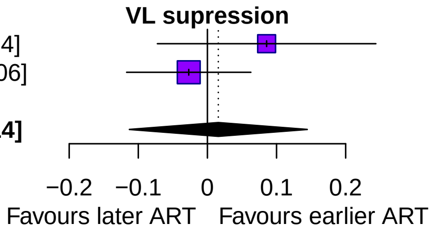

Denominator is all those who had VL measured, not all those randomised.

High CD4 counts (CD4 > 50)

Comparison A (ART <=2 weeks vs. ART >2 weeks and <=8 weeks)

	ART =<2 wks		ART 2−8 wks			
**Study**	**Events**	**Total**	**Events**	**Total**	**Weight**	**RD [95% CI]**
Amogne 1vs4and8	34	37	110	126	68.0%	0.05 [−0.06; 0.15]
Merle (RAFA)	54	72	38	49	32.0%	−0.03 [−0.18; 0.13]
**Total (95% CI)**	**88**	**109**	**148**	**175**	**100.0%**	**0.02 [−0.06; 0.11]**

Heterogeneity: $Tau^2 = 0$; $Chi^2 = 0.65$, df = 1 (P = 0.42); $I^2 = 0\%$

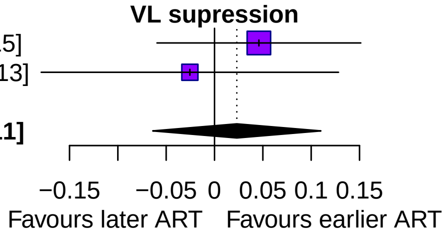

Comparison B (ART <=4 weeks vs. ART > 4 weeks)

	ART =<4 wks		ART > 4 wks			
**Study**	**Events**	**Total**	**Events**	**Total**	**Weight**	**RD [95% CI]**
Abdool Karim (SAPiT)	117	127	107	120	57.2%	0.03 [−0.04; 0.10]
Amogne 1and4vs8	81	90	63	73	30.0%	0.04 [−0.06; 0.14]
Merle (RAFA)	54	72	38	49	12.8%	−0.03 [−0.18; 0.13]
**Total (95% CI)**	**252**	**289**	**208**	**242**	**100.0%**	**0.02 [−0.03; 0.08]**

Heterogeneity: $Tau^2 = 0$; $Chi^2 = 0.52$, df = 2 (P = 0.77); $I^2 = 0\%$

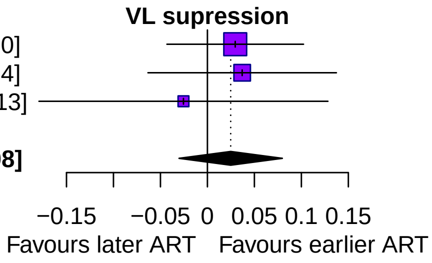

Denominator is all those who had VL measured, not all those randomised.

Loss to follow up (LFTU)

All CD4 counts

Comparison A (ART <=2 weeks vs. ART >2 weeks and <=8 weeks)

	ART =<2 wks		ART 2−8 wks			
**Study**	**Events**	**Total**	**Events**	**Total**	**Weight**	**RD [95% CI]**
Shao (THIRST)	0	35	0	35	9.3%	0.00 [−0.05; 0.05]
Blanc (CAMELIA)	6	332	6	329	65.5%	−0.00 [−0.02; 0.02]
Amogne 1vs4and8	26	163	45	315	5.8%	0.02 [−0.05; 0.08]
Merle (RAFA)	15	251	9	247	19.3%	0.02 [−0.01; 0.06]
**Total (95% CI)**	**47**	**781**	**60**	**926**	**100.0%**	**0.01 [−0.01; 0.02]**

Heterogeneity: $Tau^2 = 0$; $Chi^2 = 1.80$, df = 3 (P = 0.62); $I^2 = 0\%$

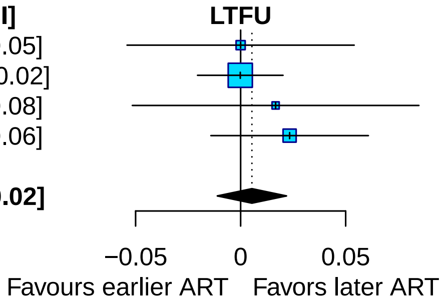

Comparison B (ART <=4 weeks vs. ART > 4 weeks)

	ART =<4 wks		ART > 4 wks			
**Study**	**Events**	**Total**	**Events**	**Total**	**Weight**	**RD [95% CI]**
Shao (THIRST)	0	35	0	35	5.1%	0.00 [−0.05; 0.05]
Blanc (CAMELIA)	6	332	6	329	20.2%	−0.00 [−0.02; 0.02]
Abdool Karim (SAPiT)	46	214	57	215	2.5%	−0.05 [−0.13; 0.03]
Havlir (STRIDE)	37	405	25	401	9.6%	0.03 [−0.01; 0.07]
Sinha	15	92	28	89	1.1%	−0.15 [−0.27; −0.03]
Manosuthi (TIME)	0	79	0	77	16.4%	0.00 [−0.02; 0.02]
Mfinanga (TB−HAART)	9	767	6	771	32.3%	0.00 [−0.01; 0.01]
Amogne 1and4vs8	50	323	21	155	3.5%	0.02 [−0.05; 0.09]
Merle (RAFA)	15	251	9	247	9.3%	0.02 [−0.01; 0.06]
**Total (95% CI)**	**178**	**2498**	**152**	**2319**	**100.0%**	**0.00 [−0.01; 0.02]**

Heterogeneity: $Tau^2 = 0.0001$; $Chi^2 = 12.18$, df = 8 (P = 0.14); $I^2 = 34\%$

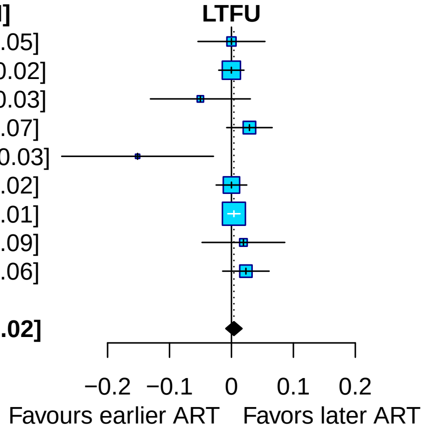

Low CD4 counts (CD4 =<50)

Comparison A (ART <=2 weeks vs. ART >2 weeks and <=8 weeks)

	ART =<2 wks		ART 2−8 wks		
**Study**	**Events**	**Total**	**Events**	**Total**	**RD [95% CI]**
Amogne 1vs4and8	10	59	12	89	0.03 [−0.08; 0.15]

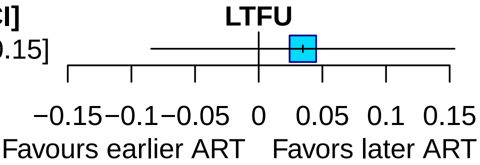

Comparison B (ART <=4 weeks vs. ART > 4 weeks)

	ART =<4 wks		ART > 4 wks			
**Study**	**Events**	**Total**	**Events**	**Total**	**Weight**	**RD [95% CI]**
Amogne 1and4vs8	17	108	5	40	12.2%	0.03 [−0.09; 0.16]
Manosuthi (TIME)	0	46	0	38	87.8%	0.00 [−0.05; 0.05]
**Total (95% CI)**	**17**	**154**	**5**	**78**	**100.0%**	**0.00 [−0.04; 0.05]**

Heterogeneity: $Tau^2 = 0$; $Chi^2 = 0.70$, df = 1 (P = 0.40); $I^2 = 0\%$

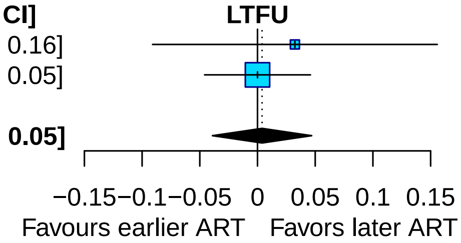

High CD4 counts (CD4 > 50)

Comparison A (ART <=2 weeks vs. ART >2 weeks and <=8 weeks)

	ART =<2 wks		ART 2−8 wks			
**Study**	**Events**	**Total**	**Events**	**Total**	**Weight**	**RD [95% CI]**
Amogne 1vs4and8	17	104	32	226	16.5%	0.02 [−0.06; 0.11]
Merle (RAFA)	15	251	9	247	83.5%	0.02 [−0.01; 0.06]
**Total (95% CI)**	**32**	**355**	**41**	**473**	**100.0%**	**0.02 [−0.01; 0.06]**

Heterogeneity: $Tau^2 = 0$; $Chi^2 = 0.00$, df = 1 (P = 0.97); $I^2 = 0\%$

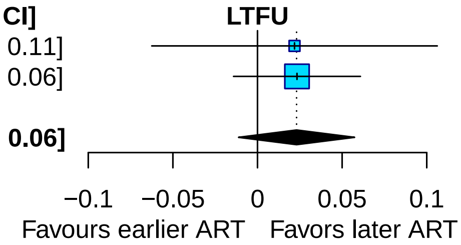

Comparison B (ART <=4 weeks vs. ART > 4 weeks)

	ART =<4 wks		ART > 4 wks			
**Study**	**Events**	**Total**	**Events**	**Total**	**Weight**	**RD [95% CI]**
Mfinanga (TB−HAART)	83	767	52	771	50.5%	0.04 [0.01; 0.07]
Amogne 1and4vs8	35	215	14	115	6.7%	0.04 [−0.04; 0.12]
Merle (RAFA)	15	251	9	247	28.6%	0.02 [−0.01; 0.06]
Manosuthi (TIME)	0	33	0	39	14.2%	0.00 [−0.05; 0.05]
**Total (95% CI)**	**133**	**1266**	**75**	**1172**	**100.0%**	**0.03 [0.01; 0.05]**

Heterogeneity: $Tau^2 = 0$; $Chi^2 = 2.33$, df = 3 (P = 0.51); $I^2 = 0\%$

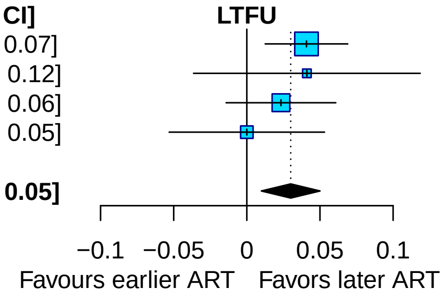

Death (sensitvity analysis)

Sensitivity analysis, where denominator is all those with ascertained outcome (ie. excluding those LTFU)

All CD4 counts

Comparison A (ART <=2 weeks vs. ART >2 weeks and <=8 weeks)

	ART =<2 wks		ART 2−8 wks			
**Study**	**Events**	**Total**	**Events**	**Total**	**Weight**	**RD [95% CI]**
Shao (THIRST)	2	35	1	35	18.6%	0.03 [−0.07; 0.12]
Blanc (CAMELIA)	46	324	63	319	29.6%	−0.06 [−0.11; 0.00]
Amogne 1vs4and8	27	137	37	270	22.9%	0.06 [−0.02; 0.14]
Merle (RAFA)	26	236	35	238	28.8%	−0.04 [−0.10; 0.02]
**Total (95% CI)**	**101**	**732**	**136**	**862**	**100.0%**	**−0.01 [−0.06; 0.05]**

Heterogeneity: $Tau^2 = 0.0016$; $Chi^2 = 6.76$, df = 3 (P = 0.08); $I^2 = 56\%$

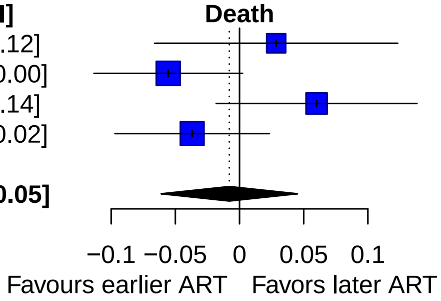

Comparison B (ART <=4 weeks vs. ART > 4 weeks)

	ART =<4 wks		ART > 4 wks			
**Study**	**Events**	**Total**	**Events**	**Total**	**Weight**	**RD [95% CI]**
Shao (THIRST)	2	35	1	35	1.9%	0.03 [−0.07; 0.12]
Blanc (CAMELIA)	46	324	63	319	5.0%	−0.06 [−0.11; 0.00]
Abdool Karim (SAPiT)	15	188	15	181	5.4%	−0.00 [−0.06; 0.05]
Havlir (STRIDE)	31	368	37	376	9.9%	−0.01 [−0.06; 0.03]
Sinha	9	88	7	62	1.7%	−0.01 [−0.11; 0.09]
Manosuthi (TIME)	6	79	5	77	2.6%	0.01 [−0.07; 0.09]
Mfinanga (TB−HAART)	19	758	21	765	65.6%	−0.00 [−0.02; 0.01]
Amogne 1and4vs8	47	273	17	134	3.3%	0.05 [−0.03; 0.12]
Merle (RAFA)	26	236	35	238	4.7%	−0.04 [−0.10; 0.02]
**Total (95% CI)**	**201**	**2349**	**201**	**2187**	**100.0%**	**−0.01 [−0.02; 0.01]**

Heterogeneity: $Tau^2 = 0$; $Chi^2 = 7.54$, df = 8 (P = 0.48); $I^2 = 0\%$

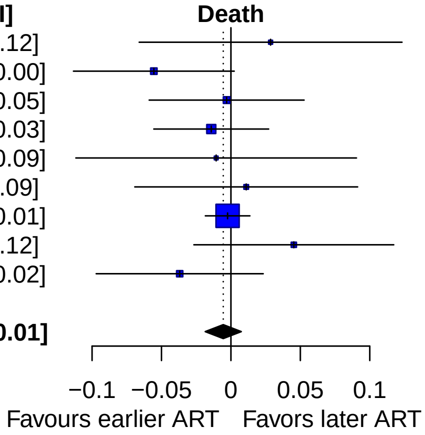

Low CD4 counts (CD4 =<50)

Comparison A (ART <=2 weeks vs. ART >2 weeks and <=8 weeks)

	ART =<2 wks		ART 2−8 wks			
**Study**	**Events**	**Total**	**Events**	**Total**	**Weight**	**RD [95% CI]**
Amogne 1vs4and8	16	59	21	89	100.0%	0.04 [−0.11; 0.18]
Blanc (CAMELIA)	39	.	51	.	0.0%	
**Total (95% CI)**	**55**	**59**	**72**	**89**	**100.0%**	**0.04 [−0.11; 0.18]**

Heterogeneity: Tau^2 = NA; Chi^2 = 0.00, df = 0 (P = NA); I^2 = NA%

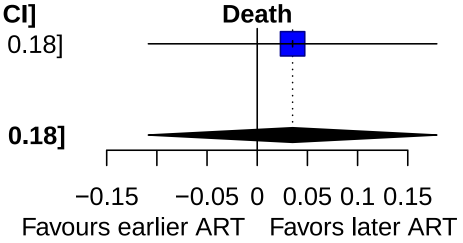

Comparison B (ART <=4 weeks vs. ART > 4 weeks)

	ART =<4 wks		ART > 4 wks			
**Study**	**Events**	**Total**	**Events**	**Total**	**Weight**	**RD [95% CI]**
Abdool Karim (SAPiT)	3	37	7	35	29.3%	−0.12 [−0.28; 0.04]
Havlir (STRIDE)	14	.	24	.	0.0%	
Amogne 1and4vs8	27	108	10	40	30.0%	0.00 [−0.16; 0.16]
Blanc (CAMELIA)	39	.	51	.	0.0%	
Manosuthi (TIME)	4	46	5	38	40.7%	−0.04 [−0.18; 0.09]
**Total (95% CI)**	**87**	**191**	**97**	**113**	**100.0%**	**−0.05 [−0.14; 0.03]**

Heterogeneity: Tau^2 = 0; Chi^2 = 1.14, df = 2 (P = 0.56); I^2 = 0%

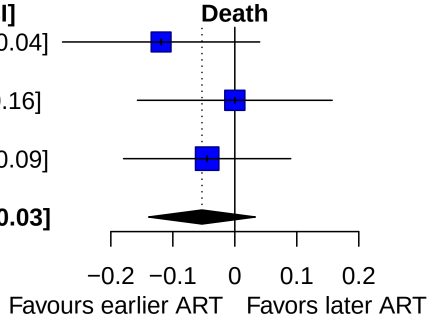

High CD4 counts (CD4 > 50)

Comparison A (ART <=2 weeks vs. ART >2 weeks and <=8 weeks)

	ART =<2 wks		ART 2−8 wks			
**Study**	**Events**	**Total**	**Events**	**Total**	**Weight**	**RD [95% CI]**
Amogne 1vs4and8	11	104	16	226	47.5%	0.03 [−0.03; 0.10]
Blanc (CAMELIA)	7	.	12	.	0.0%	
Merle (RAFA)	26	236	35	238	52.5%	−0.04 [−0.10; 0.02]
**Total (95% CI)**	**44**	**340**	**63**	**464**	**100.0%**	**−0.00 [−0.07; 0.07]**

Heterogeneity: $Tau^2 = 0.0016$; $Chi^2 = 2.45$, df = 1 (P = 0.12); $I^2 = 59\%$

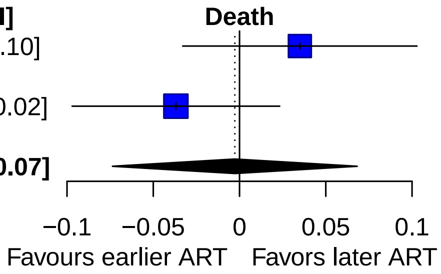

Comparison B (ART <=4 weeks vs. ART > 4 weeks)

	ART =<4 wks		ART > 4 wks			
**Study**	**Events**	**Total**	**Events**	**Total**	**Weight**	**RD [95% CI]**
Abdool Karim (SAPiT)	12	177	8	180	16.8%	0.02 [−0.02; 0.07]
Havlir (STRIDE)	17	.	13	.	0.0%	
Mfinanga (TB−HAART)	19	684	21	719	54.8%	−0.00 [−0.02; 0.02]
Amogne 1and4vs8	20	215	7	115	12.0%	0.03 [−0.03; 0.09]
Blanc (CAMELIA)	7	.	12	.	0.0%	
Merle (RAFA)	26	236	35	238	11.4%	−0.04 [−0.10; 0.02]
Manosuthi (TIME)	2	33	0	39	5.1%	0.06 [−0.03; 0.15]
**Total (95% CI)**	**103**	**1345**	**96**	**1291**	**100.0%**	**0.01 [−0.02; 0.03]**

Heterogeneity: $Tau^2 = 0.0001$; $Chi^2 = 5.06$, df = 4 (P = 0.28); $I^2 = 21\%$

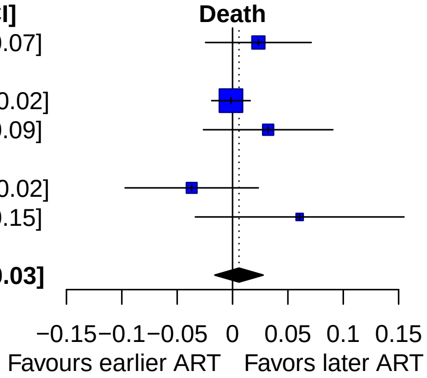

Supplement: Supplementary file 4 — Figures S1. Forest plots for all outcomes, for all participants and by CD4 count. [file JIA2-24-e25772-s003.pdf]
